# Psychosocial effects of the COVID-19 pandemic and lockdown on university students: Understanding apprehensions through a phenomenographic approach

**DOI:** 10.1371/journal.pone.0251641

**Published:** 2021-05-13

**Authors:** Sumbal Shahbaz, Muhammad Zeshan Ashraf, Rubeena Zakar, Florian Fischer, Muhammad Zakria Zakar

**Affiliations:** 1 Department of Public Health, Institute of Social and Cultural Studies, University of the Punjab, Lahore, Pakistan; 2 Department of Architecture, University College of Art and Design, University of the Punjab, Lahore, Pakistan; 3 Institute of Public Health, Charité –Universitätsmedizin Berlin, Berlin, Germany; 4 Institute of Gerontological Health Services and Nursing Research, Ravensburg-Weingarten University of Applied Sciences, Weingarten, Germany; 5 University of Okara, Okara, Pakistan; Medical University Innsbruck, AUSTRIA

## Abstract

Limited evidence exists to help understand the experiences of university students in relation to the long-term lockdown due to the COVID-19 pandemic. For that reason, we conducted a study using a phenomenographic approach in order to understand how university students perceive COVID-19 and the associated lockdown. Data were collected from 25 students in Pakistan. They were asked to demonstrate the psychological effects of the COVID-19 pandemic and lockdown in illustrations. In addition, in-depth interviews were conducted with these students, to gain further insights into their perspectives on the psychosocial effects of the COVID-19 pandemic. The analysis revealed four interlinked directions for understanding students’ experiences. These themes were: 1) escape into peace, 2) hope for personal freedom, 3) fear of becoming a victim of COVID-19, and 4) concerns regarding education, future career, and opportunities. All four themes were analyzed and condensed into an outcome space, which further gathers the perceptions of students under one theme as “Hope for life while paradoxically living with fear”. Studying the psychological impact of the COVID-19 pandemic and lockdown on students not only highlighted their concerns, but also emphasized the importance of starting regular psychological evaluations and stress-releasing sessions, along with online education to overcome growing depression.

## Background

COVID-19 is a public health emergency of international concern [1]. Its symptoms range from minor to severe pneumonia and Acute Respiratory Disease Syndrome (ARDS), to septic shock, and in some cases multiple organ failure [2]. A rapid increase in the number of suspected and confirmed cases was verified in various countries, which proved this pandemic to be more easily transmittable than SARS. About 22% of reported COVID-19 cases and more than 80% of reported COVID-19 deaths have been among older adults. Specific comorbidities (e.g., diabetes, chronic underlying lung or heart disease, immune-compromising conditions/treatments), low socioeconomic status (i.e., poor access to good-quality healthcare facilities), and high population density all worsen the situation [3]. Moreover, there is great unpredictability among all age groups related to whom the disease will affect more severely and how long it will last. This enforces feelings of ambiguity and insecurity, as well as amplifying stress levels among communities [4]. Therefore, it is a global challenge and threat to public health security [5, 6].

Due to this pandemic situation, not only are health-care providers overburdened, but public health departments all over the world initially recommended physical distancing and later on massive lockdowns due to the ongoing spread of the virus within the public [7]. Lockdown is a term substitute for mass quarantine, which could include the order to stay at home, thus limiting or entirely obliterating the movement of individuals. Hence, the pandemic poses psychological pressure not only on COVID-19 patients and their relatives, but also on the healthy population [8].

When implemented as a control strategy affecting a massive number of people, lockdowns lead to challenges such as the questions of how long these actions are needed and how to separate suspected cases from healthy individuals [9]. In addition, tough regulatory measures, such as restraining social life in community spaces by closing down public and private organizations, temporarily closing educational institutes and businesses for non-critical goods, and strict curfews imposed with the help of the police or army have major adverse socio-economic and psychosocial implications [10].

The mental health of students at universities and professional institutes is anticipated to be influenced by the pandemic and its associated political actions in terms of severe quarantine measures and postponements of the opening of educational institutes throughout the world. There are studies available on the psychological and socio-economic influences of the COVID-19 pandemic on health-care personnel, adults, and children [11–13]. However, until now no comprehensive qualitative research has been conducted on the mental wellbeing of young university students facing the pandemic and lockdowns to gain insight into their fears and reservations [14].

When considering the phenomenon of physical distancing or quarantining, it is essential to realize that it comes with a significant personal cost and disrupts the physical and mental wellbeing of those facing it. It involves reduced social contact with friends and family, as well as confinement to a specific space. Even though these precautionary measures are essential for reducing the pressure on health-care systems, the troublesome part is that extended home quarantine due to any infectious outbreak might damage the physical and mental wellbeing of people facing it [7, 15]. It can decrease participation in physical and satisfying activities as well as intensifying stress levels caused by social isolation [16].

As Pakistan is among the countries most affected by COVID-19 (according to statistics from July 2020), the government is conscious of the urgent need for action. Hence, a nationwide lockdown was initiated on March 23, 2020 [6], and educational institutes were closed on March 14, 2020. This had an impact on all students in terms of decreases in physical activity, increased screen time, weight gain, and uncertainty about higher education and career development [17].

From the beginning of the COVID-19 pandemic, feelings of anxiety and fear were common among the community [18]. The misery of anticipating death nearby and the hope of surviving this difficult period were common. Pandemics trigger a broad range of psychiatric disturbances, such as anxiety, panic attacks, insomnia, bad temper, and melancholy or depression. All of these appear to have a large impact on university students [19]. Therefore, this study aims to understand the psychosocial effects of COVID-19 and the associated lockdown on university students in Pakistan. These effects are witnessed through investigating artistic illustrations which can not only convey an insider’s perspective on Pakistani students but can also provide an innovative method for collecting profound qualitative data during specific periods, such as a pandemic. The study narrates a story of socially constrained youngsters and provides an insight into their feelings and expectations. Thus, it allows us to define diverse ways in which this practice generates meaning.

## Materials and methods

### Study design

To gain an insight into the practices and apprehensions of university students during the COVID-19 lockdown, a phenomenographic methodology was adopted. Phenomenography is a type of qualitative methodology that is used to define disparities among people’s understandings and conceptualizations in their own way [20]. It defines the ways in which a set of people constrained by specific circumstances understand their experience(s), and readers are provided with possible variations in the ways of understanding the same phenomena. The aim of this methodology is to determine various understandings or knowledge about certain phenomena. The researcher is devoted to developing a philosophical meaning in addition to being cautious about unfolding the means by which experience is assumed [21].

Phenomenographic investigations are usually expressed as categories of description or themes which portray the core meaning of concepts and the ways in which they could be analyzed, labelled, and understood. It could also be considered as an abstract tool to symbolize and summarize different understandings of phenomena identified through the analysis of data. An outcome space is then used to represent the logical relationships between different identified themes. According to Marton [20], an outcome space is an empirical map of the different ways in which people perceive and understand various aspects of the world around them.

This study uses illustrations to express understandings of lockdown and COVID-19. Although this is an uncommon method, it is an acknowledged research method in phenomenography [21]. The illustrations present a creative manifestation, revealing the associations made by university students in Pakistan facing pandemic restrictions and experiencing lockdowns. Each illustration in our study conveyed the associations of the participant and his or her understanding of the phenomena [20, 21]. Illustrations served as the data, which symbolized mutual reflections of the meaning.

### Sample

Participants were selected randomly from different departments of the University College of Art and Design at the University of the Punjab in Lahore, Pakistan. The selection of these students is justified because: 1) art students studying at this institution come from all over Pakistan, 2) they have diverse socioeconomic backgrounds, and 3) they are skilled enough to convey their message or feeling in the form of an illustration. Thirty-two students volunteered to take part in the study. However, only 25 of them were able both to hand in an illustration and be available for an online-based qualitative interview afterwards.

### Data collection

Participating students were asked to define their understanding of the ways in which they acknowledge their feelings related to the COVID-19 pandemic and lockdown through illustrations such as drawings. The artists were free in their choice of art technique, e.g. color/charcoal pencils, pastels, or watercolors. After finishing the illustration, each participant was asked to explain the meaning of his or her drawing in an online session.

Further probing questions were asked related to the illustrations. For example, in Fig 7 the participant has illustrated the stress and terror around him by using the color red and hope with the color yellow. The probing questions in this case were: 1) “What actually terrifies you?” and 2) “Please define the hope that supports you to survive.”

After taking written consent from the participants, these explanations were recorded. Afterwards, the statements were transcribed and, in conjunction with the associated illustrations, served as information for the phenomenographic investigation.

### Data analysis

The illustrations, supported by additional verbatim clarifications provided by the students, were analyzed. We compared, grouped, labeled, and contrasted the understandings presented in painted form in order to establish categories of description. In the next step, we condensed the categories into overarching themes. These themes depict the core connotation of each concept and emphasize variations and similarities among participants’ understanding of the phenomenon. This led to four categories of description:

1. Escape into peace
2. Hope for personal freedom: A world without fear of COVID-19 and lockdowns
3. Fear of being a victim of COVID-19
4. Concerns regarding education, future career, and opportunities

The final themes reflected the diverse understandings of surviving a pandemic and lockdown, which were contextually elucidated in the form of a mutual understanding referred as the outcome space.

### Ethical considerations

The research was approved by the Ethical and Research Board of the Department of Public Health, University of the Punjab, Lahore (D/577/ERBPH/ISCS). Individual written consent to contribute was attained after rapport building and explaining the study to the participants.

## Results

Categories of description recognized through the data–i.e., concepts about surviving the pandemic and lockdown–were analyzed. The conceptual framework originated from respondents’ personal feelings and experiences, which represent a mutual understanding of these phenomena.

### Escape into peace

**Fig 1:**
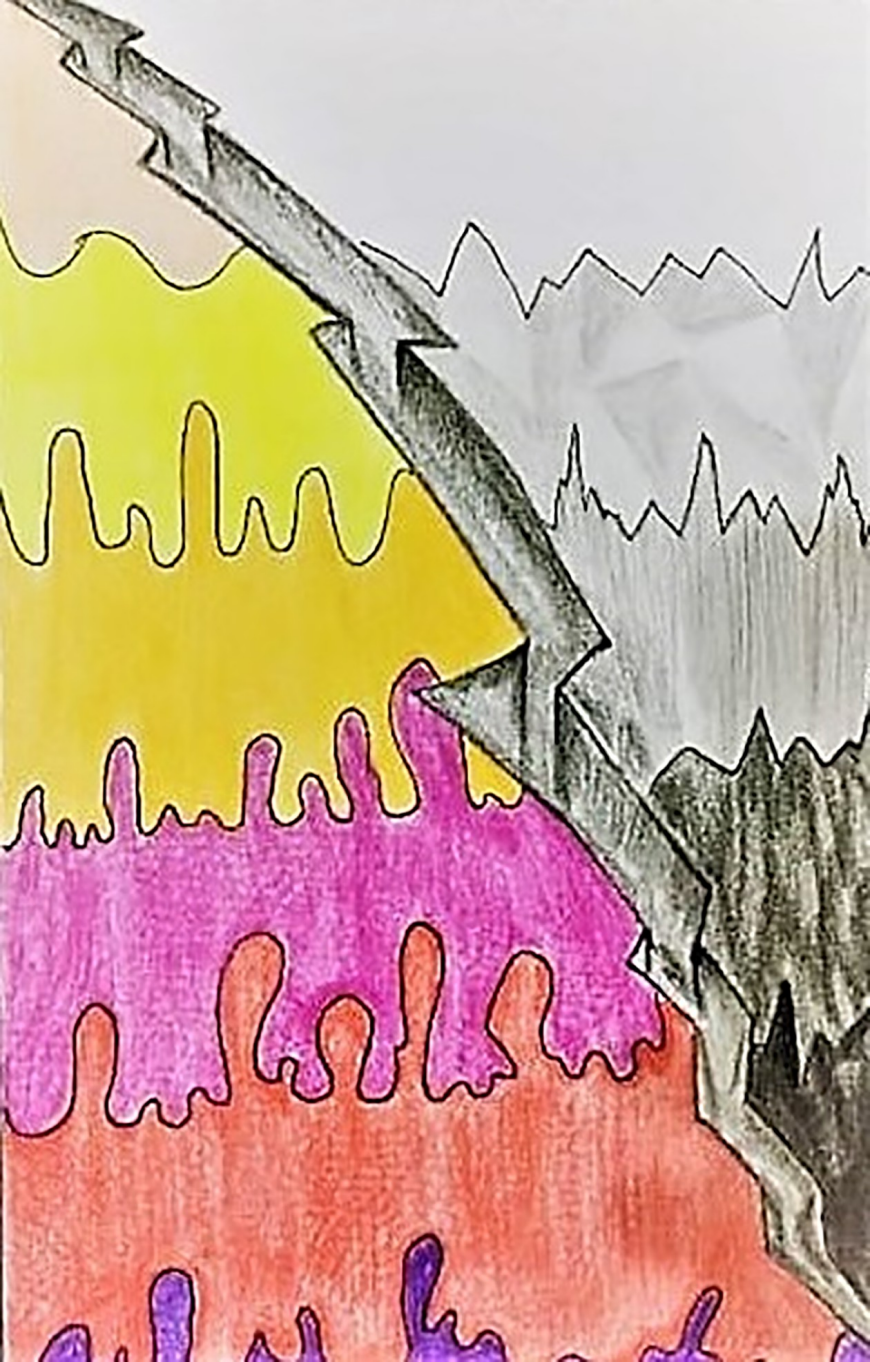
Illustration “escape into peace” (1).

**Fig 2:**
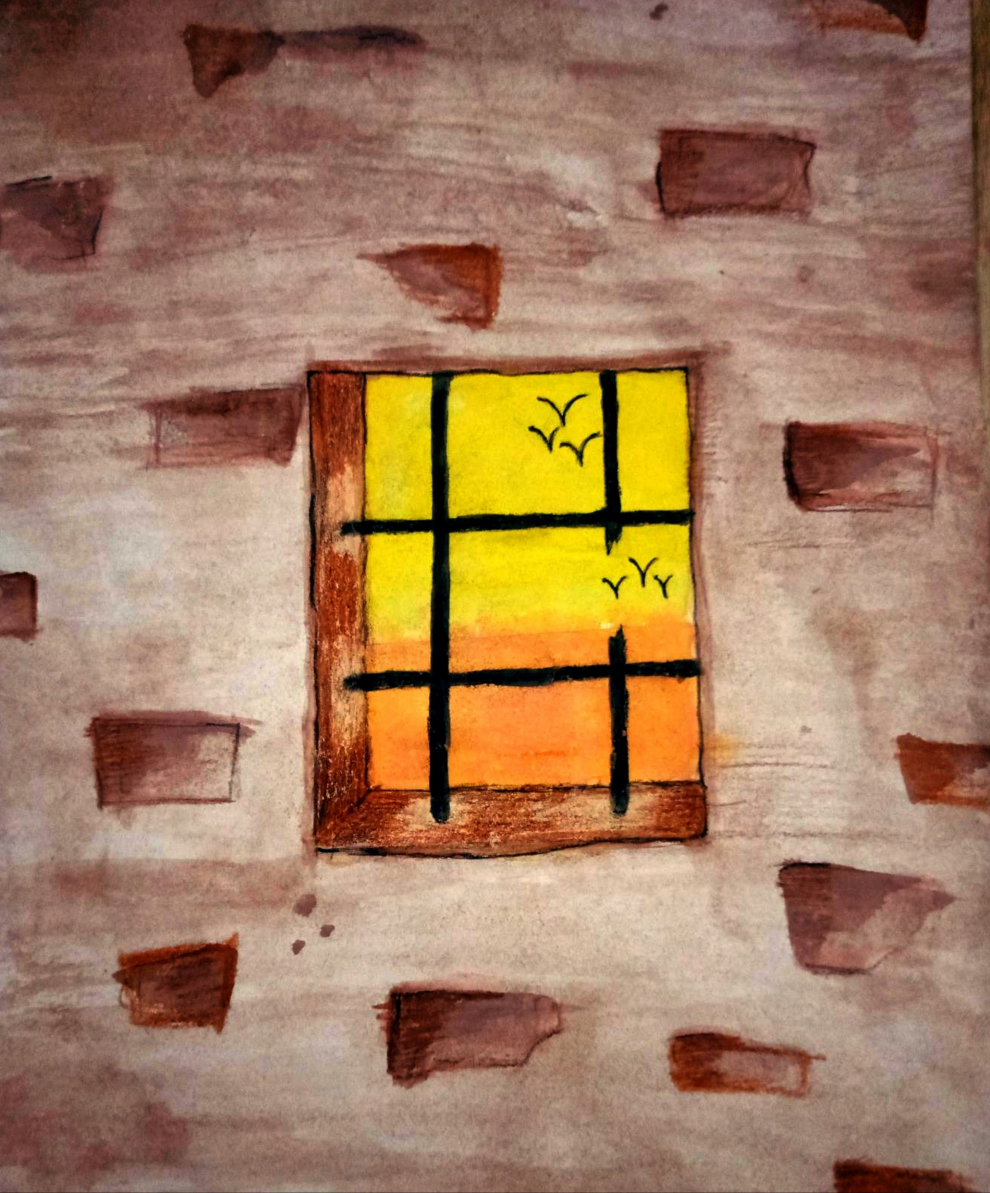
Illustration “escape into peace” (2).

Being at peace or achieving harmony appears to be the fundamental basis of hope and positivity. One student described her feelings as “quarantine and lockdowns due to corona are not only breaking us apart socially but also psychologically. Eventually causing the fear of death and at the same time having a hope that it will end someday, and we will be out and free with happy souls as we were before” (Fig 1). The colors in the picture depict the colors of life overcoming the darkness of fear and death–emphasizing the hope for a better and more normal future. Another participant (Fig 2) expressed his feelings and explained:

“We often get weak and stressed out, considering there is no way out of the situation. But all we need is to calm ourselves and believe that everything will soon be fine. Better is always on its way and in the end peace always prevails.”

The broken window grills can be seen as a way to escape from the prison–which is a symbol for the lockdown–into a world full of freedom, indicated by the birds outside.

**Fig 3:**
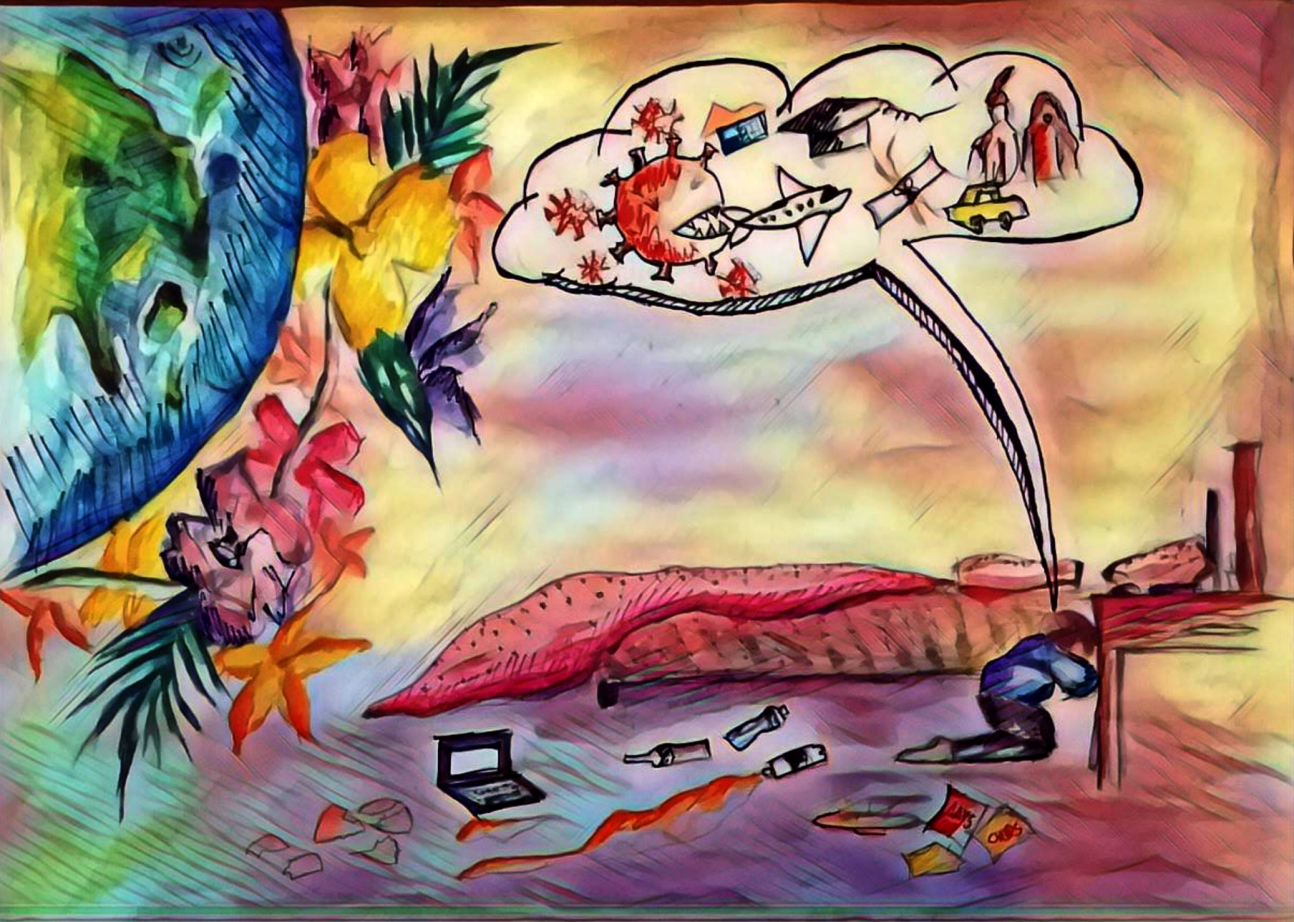
Illustration “escape into peace” (3).

As a qualitative study explores the world through the participants’ perspectives, one of the participants labelled the pandemic and lockdown as a blessing in disguise. According to him, “this virus has caged the selfish desires of mankind. While our dreams have blown away with the autumn of pandemic, animals and nature are blooming” (Fig 3). The participant considered this time to be a feast for nature, which is constantly being destroyed by human beings. Now, when humans are forced to stay indoors, nature is living free of fear and the atmosphere is healing itself. Depression has reached its peak, but so has repression.

**Fig 4:**
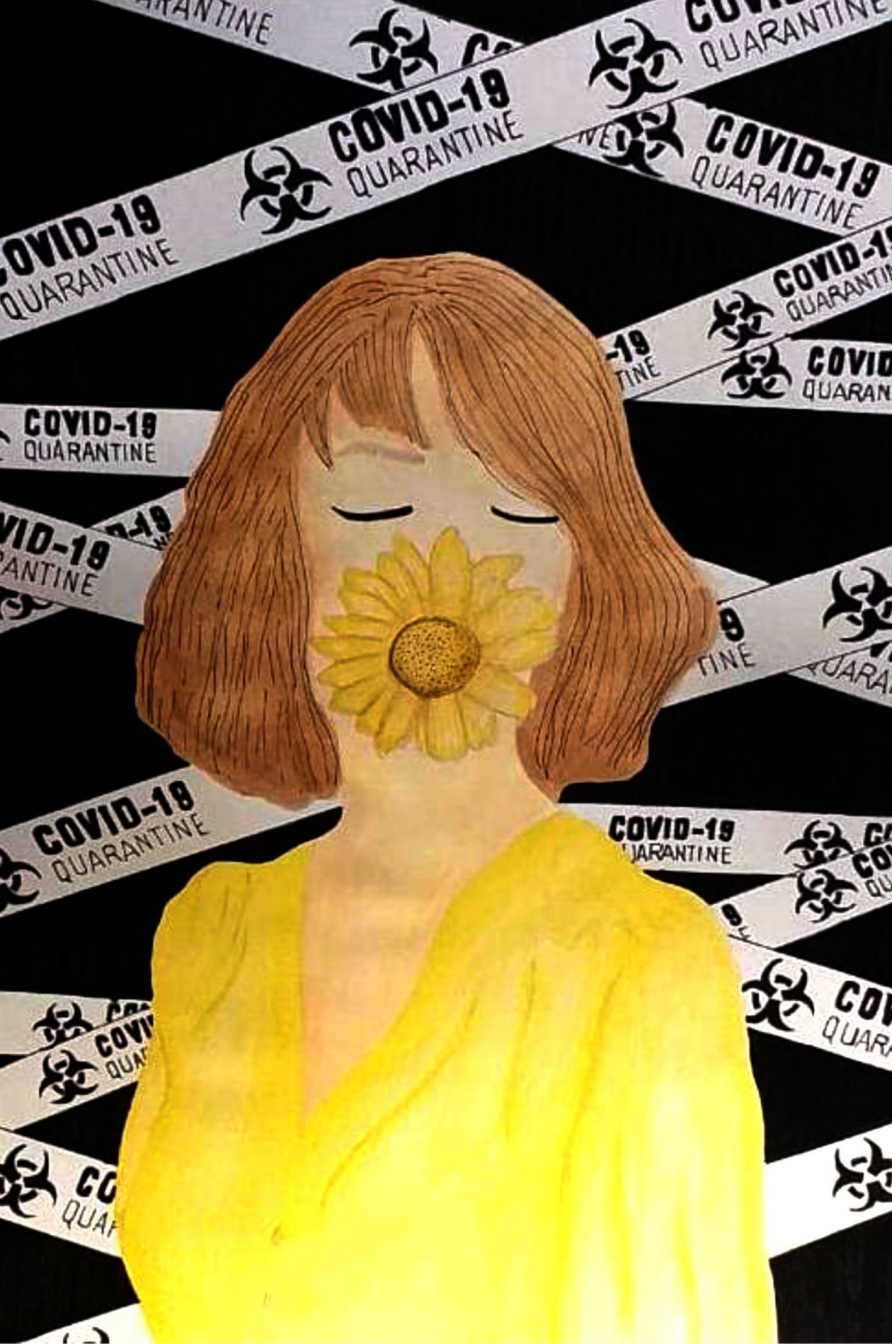
Illustration “escape into peace” (4).

Similarly to her illustration, another female participant reflected on optimism, hope and assurance (Fig 4). The sunflower covering the mouth is a replacement for the mouth-and-nose facemask and should symbolize bliss, whereas the yellow dress predicts the eradication of the COVID-19 pandemic. Her closed eyes indicate the wishes and prayers for affected ones, while short hair represents damage to the stability of the earth and the economy. Obscurity and trouble are represented by the black background. The whole illustration delivers the message that hope prevails in times of desperation.

**Fig 5:**
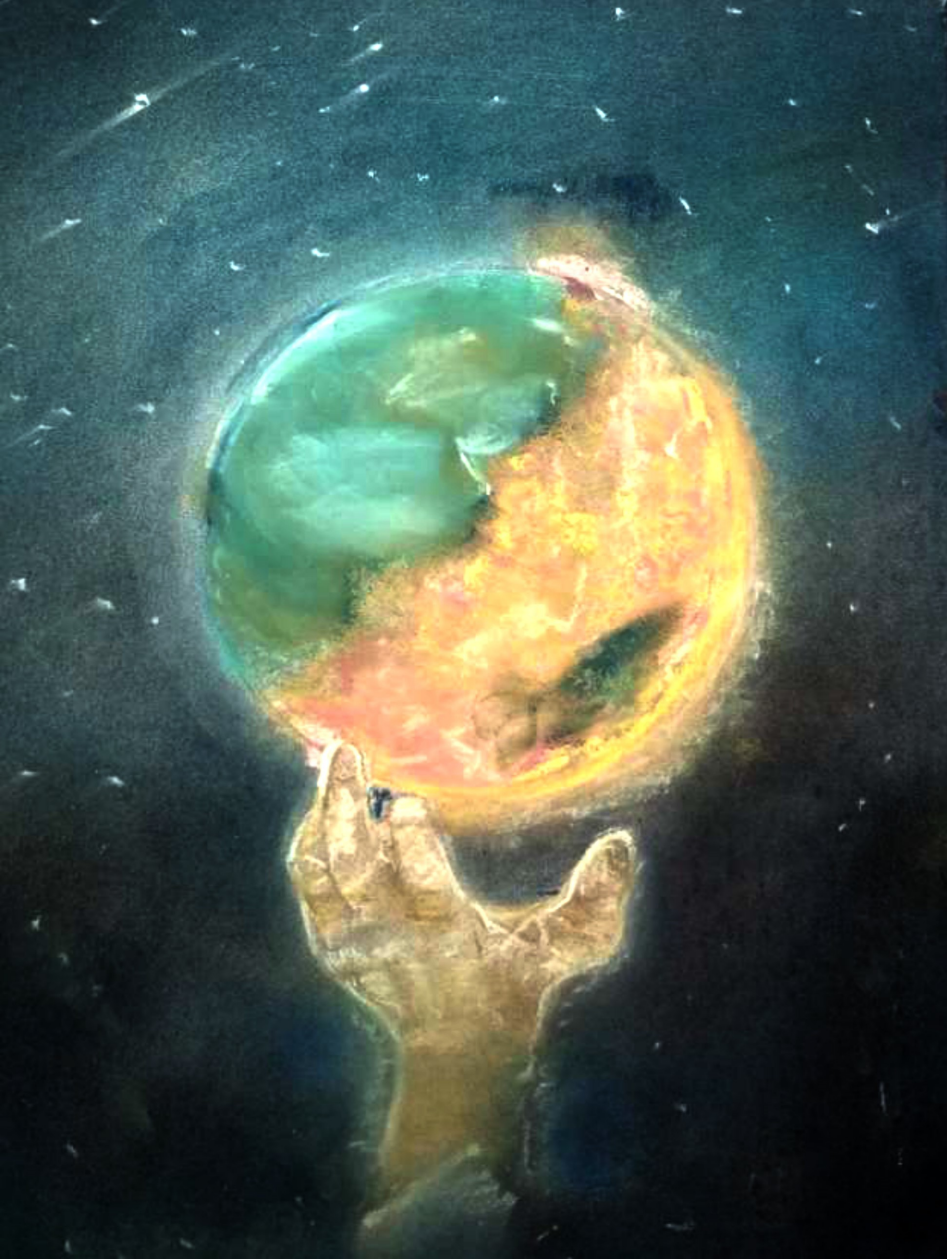
Illustration “escape into peace” (5).

The final illustration under this theme (Fig 5) shows the globe on fire, representing the destruction caused by humans through their actions–depicted by the human hand. The fire is trying to reach the peaceful side, which is enduring with fear. But this fear could be used as a weapon to fight the evil. The participant considers that humans have dragged themselves into this war. Therefore, it is time to correct the mistakes that humankind has made, such as ruining peace and poisoning the atmosphere of our planet. These participants were coping actively with the situation and had a firm belief that things would soon be back to normal.

### Hope for personal freedom: A world without fear of COVID-19 and lockdowns

**Fig 6:**
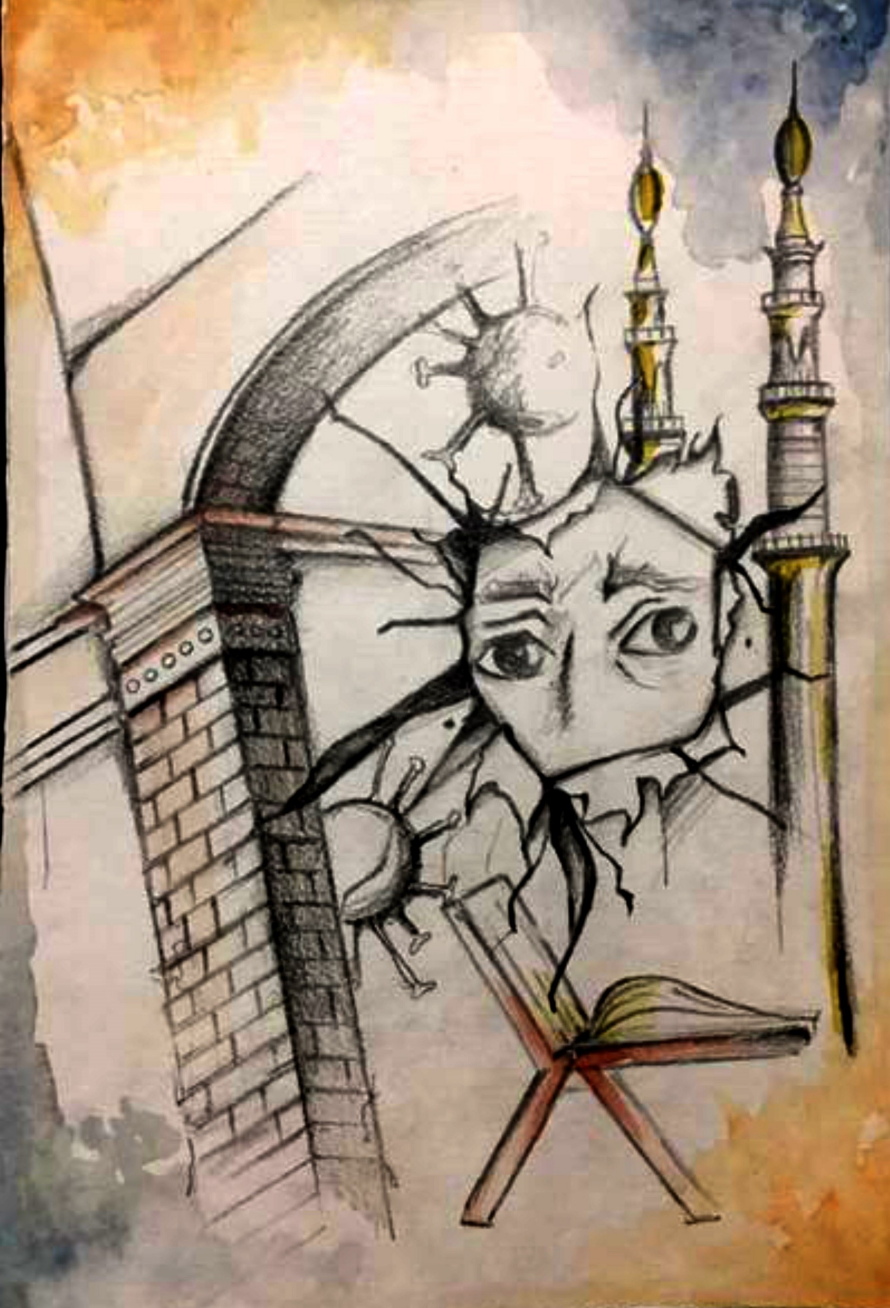
Illustration “hope for personal freedom” (1).

Hope is the key to survival and to overcoming any difficult situation, irrespective of the misery one is facing [22]. According to the illustrator of Fig 6, “hope comes in the form of faith in the almighty or optimism about a life free of restrictions, even though death could be anticipated nearby.” In her illustration, she has depicted fear about her hopes and dreams being torn apart, as well as her faith that only turning towards the Almighty can undo the destruction this disease has caused.

**Fig 7:**
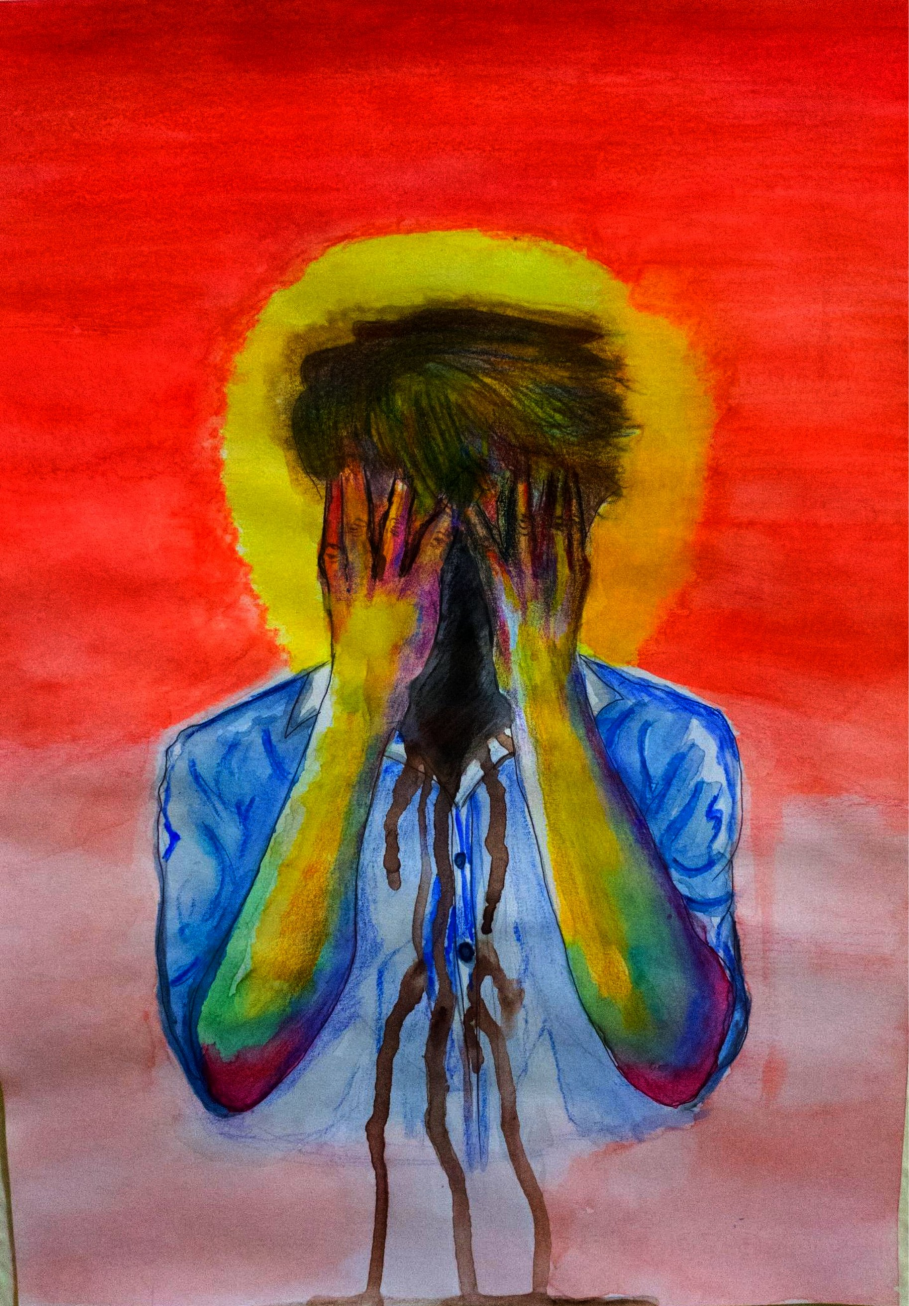
Illustration “escape into peace” (2).

Another respondent described life in quarantine as a new and unexpected way of living. He said that, as a youngster, he felt miserable, being locked in the house without meeting friends or members of his social circle. In his illustration (Fig 7), he has shown himself colored blue. This symbolizes a person who has been happy but now is just pretending to be calm, although in reality he is stressed. The red color in the background depicts the terror and tension around him, while the yellow color refers to the hope and positivity that is allowing him to survive.

**Fig 8:**
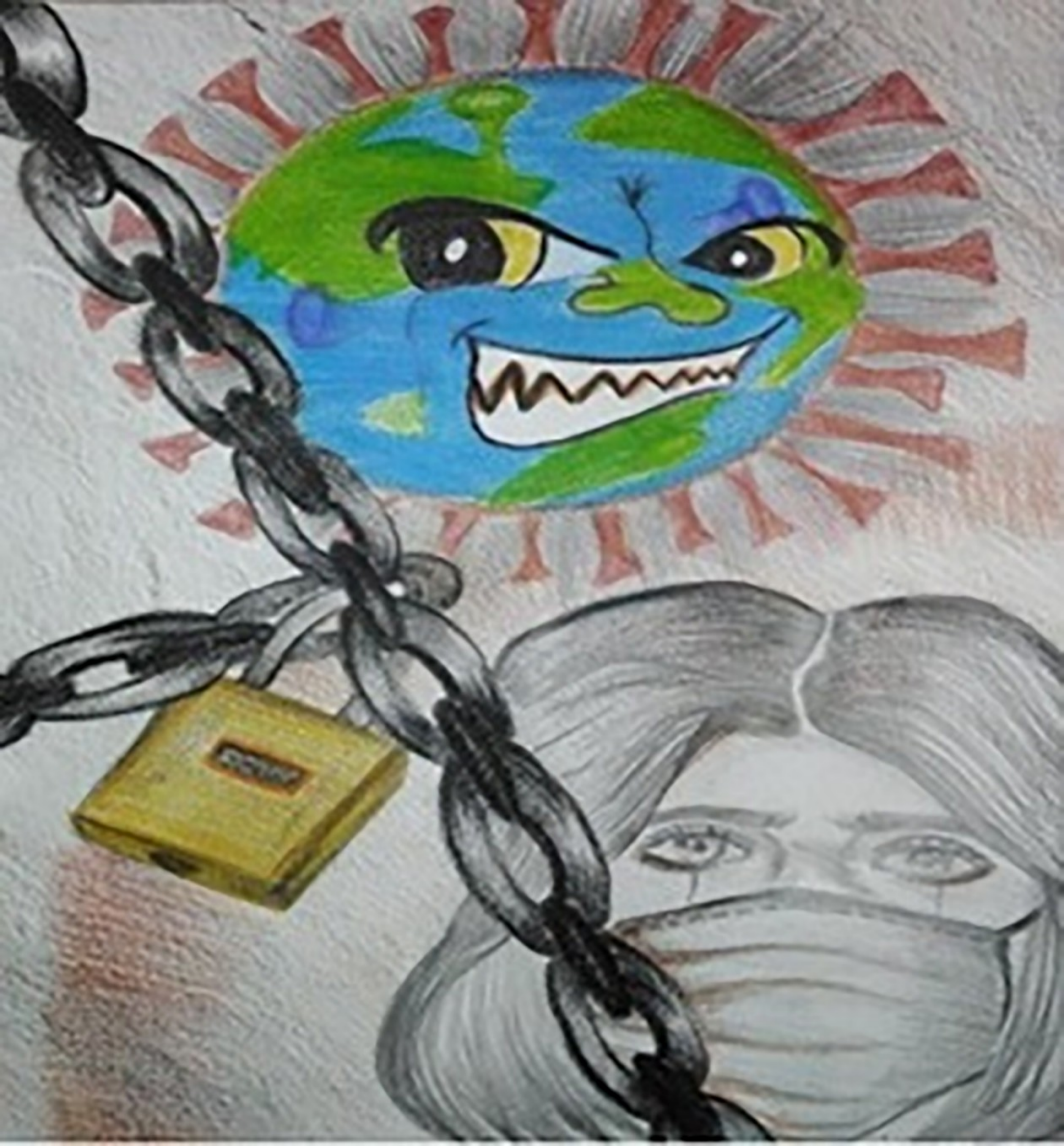
Illustration “escape into peace” (3).

**Fig 9:**
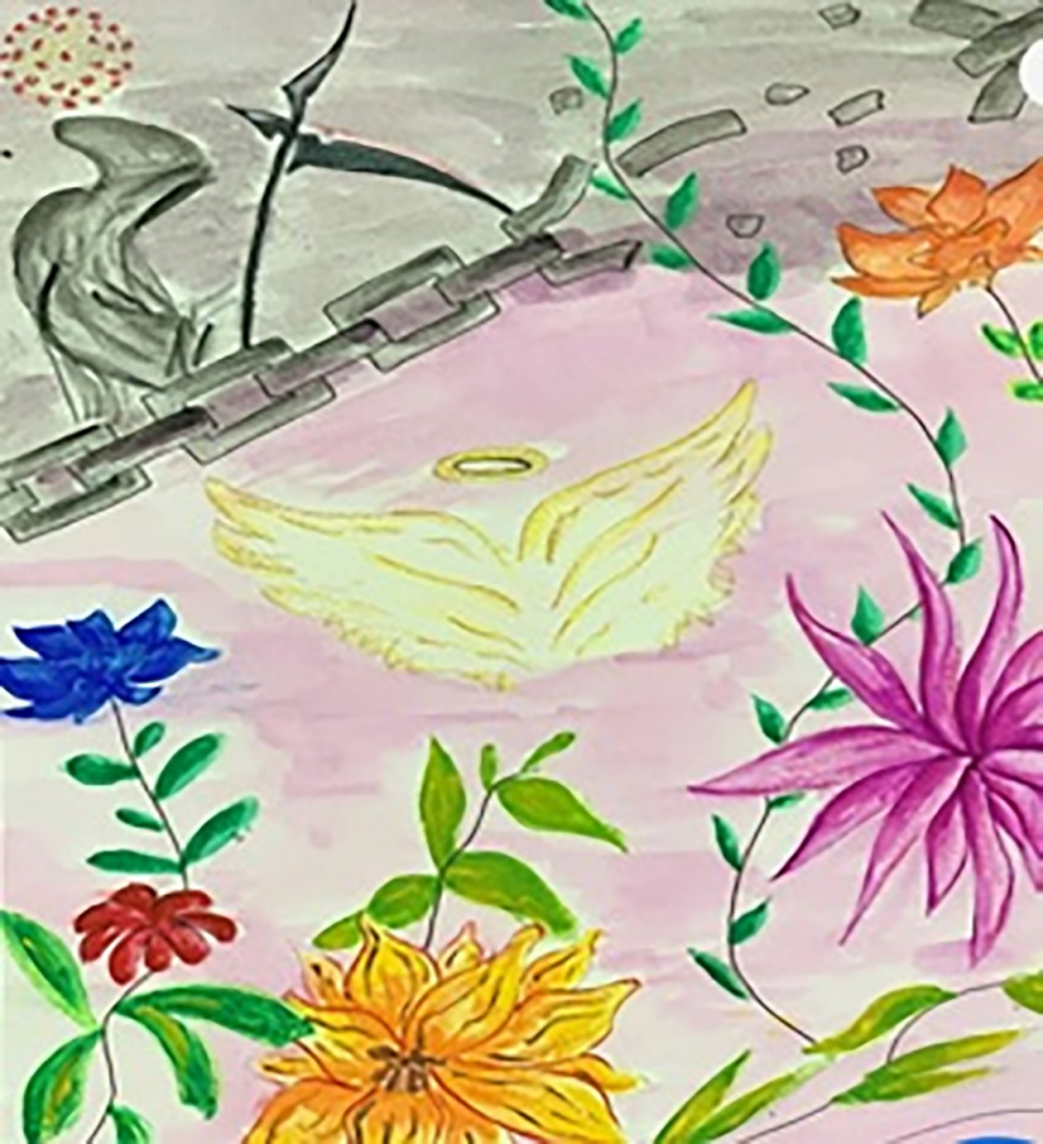
Illustration “escape into peace” (4).

Fig 8 elucidates that the earth–illustrated to appear similar to the COVID-19 virus–has been locked up by this invisible deadly enemy which has brought chaos to our lives and triggered economic crises all over the world. The girl wearing a mask represents the effort of trying to save lives and hope that the situation will soon be over. On the other hand, few students showed a high level of optimism. Fig 9 represents strong hope and faith, which the study participants wanted to spread around. These flowers represent the hope of freedom, growth, and positivity, while the angel’s wings are people trying to save others.

**Fig 10:**
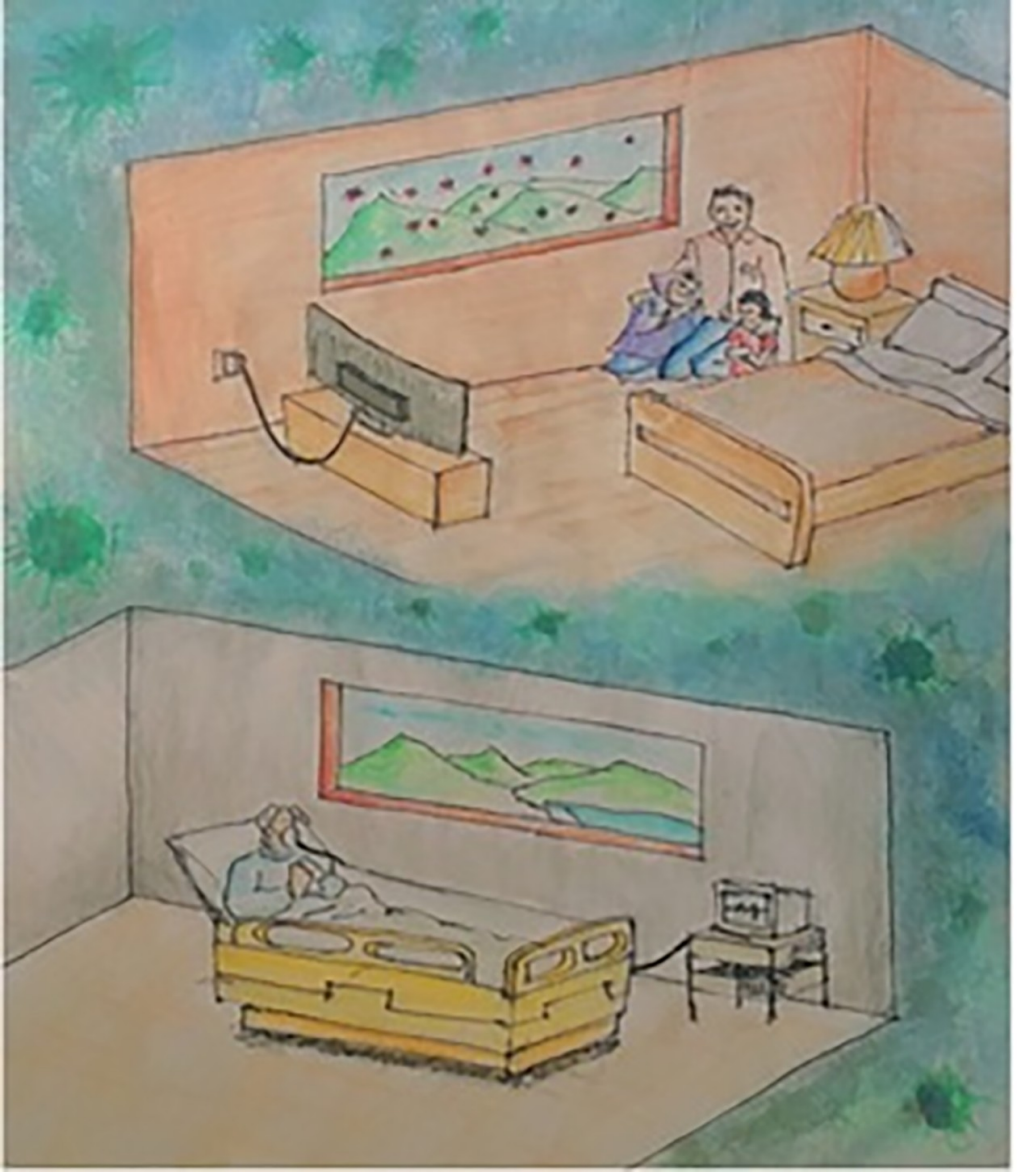
Illustration “escape into peace” (5).

**Fig 11:**
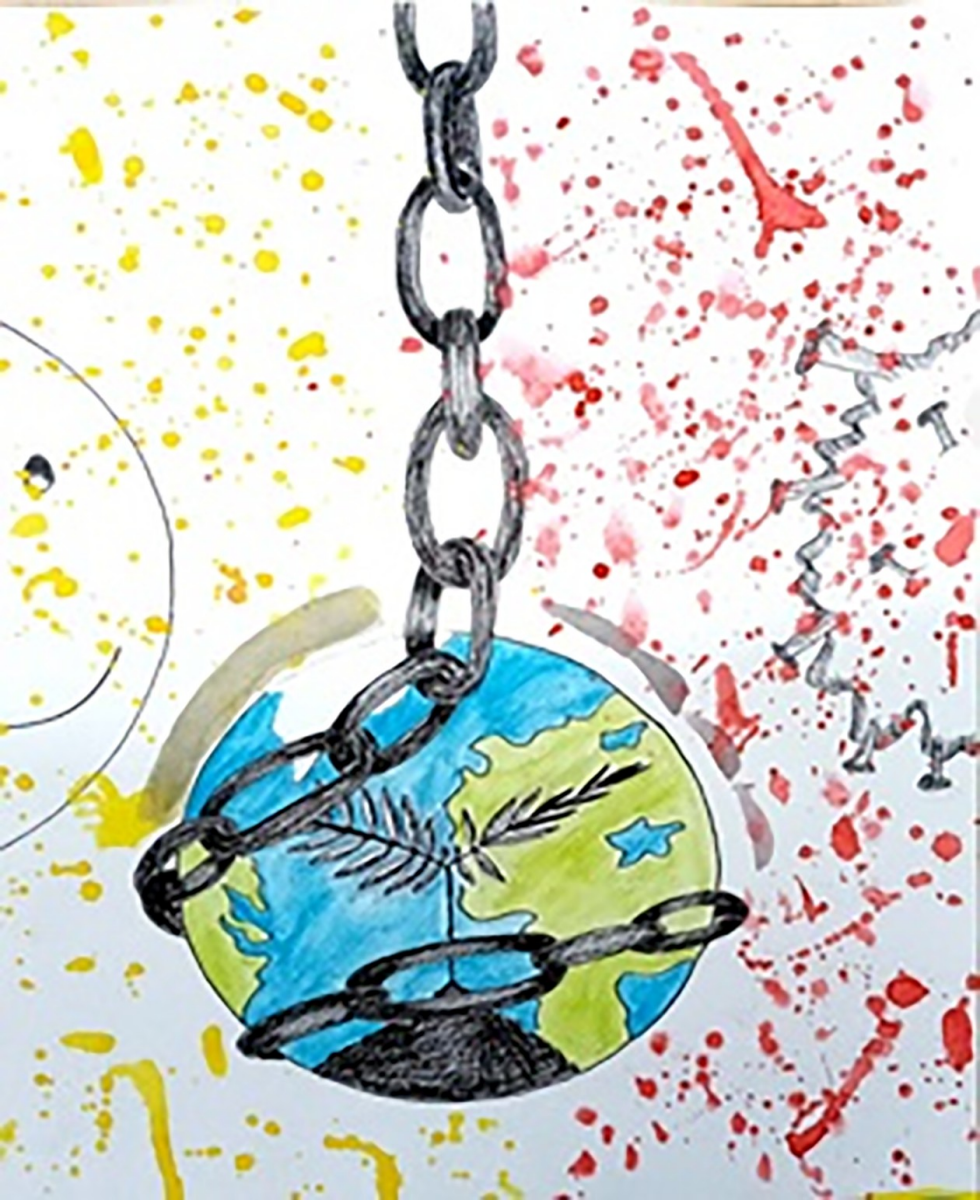
Illustration “escape into peace” (6).

Another illustration (Fig 10) portrays the hope among COVID-19 affected and non-affected people. According to this participant, sufferers are quarantined in loneliness, hoping to live normally and feel the beauty outside again as soon as possible, while healthy people are quarantined with their loved ones, stressed about their safety and assuming that outside there is death everywhere. Both now understand the beauty of life without fear and how much they took it for granted before. Fig 11 shows the earth as bound with chains and turned into a plant that does not want to be touched anymore. The years of misuse have made it angry towards humankind, but still there is hope and peace on the left-hand side, while death reigns on the other side. The greyish layer stands for the ozone layer, which is rejuvenating.

These illustrations portray misery, articulating with faith in the return of a free life (without the chains that frequently occur in the drawings). Thus, they depict intermittent active and positive coping techniques in participants’ minds.

### Fear of being a victim of COVID-19

**Fig 12:**
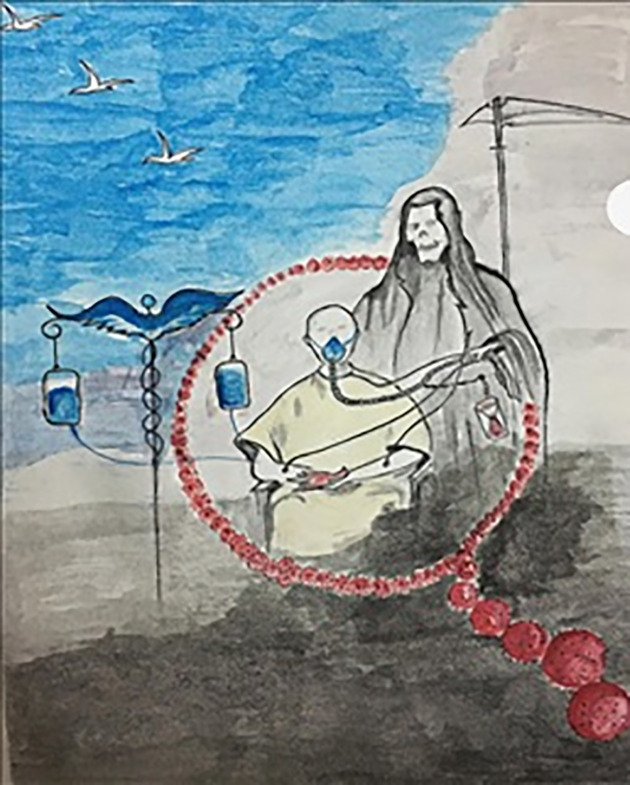
Illustration “fear of being a victim of COVID-19” (1).

**Fig 13:**
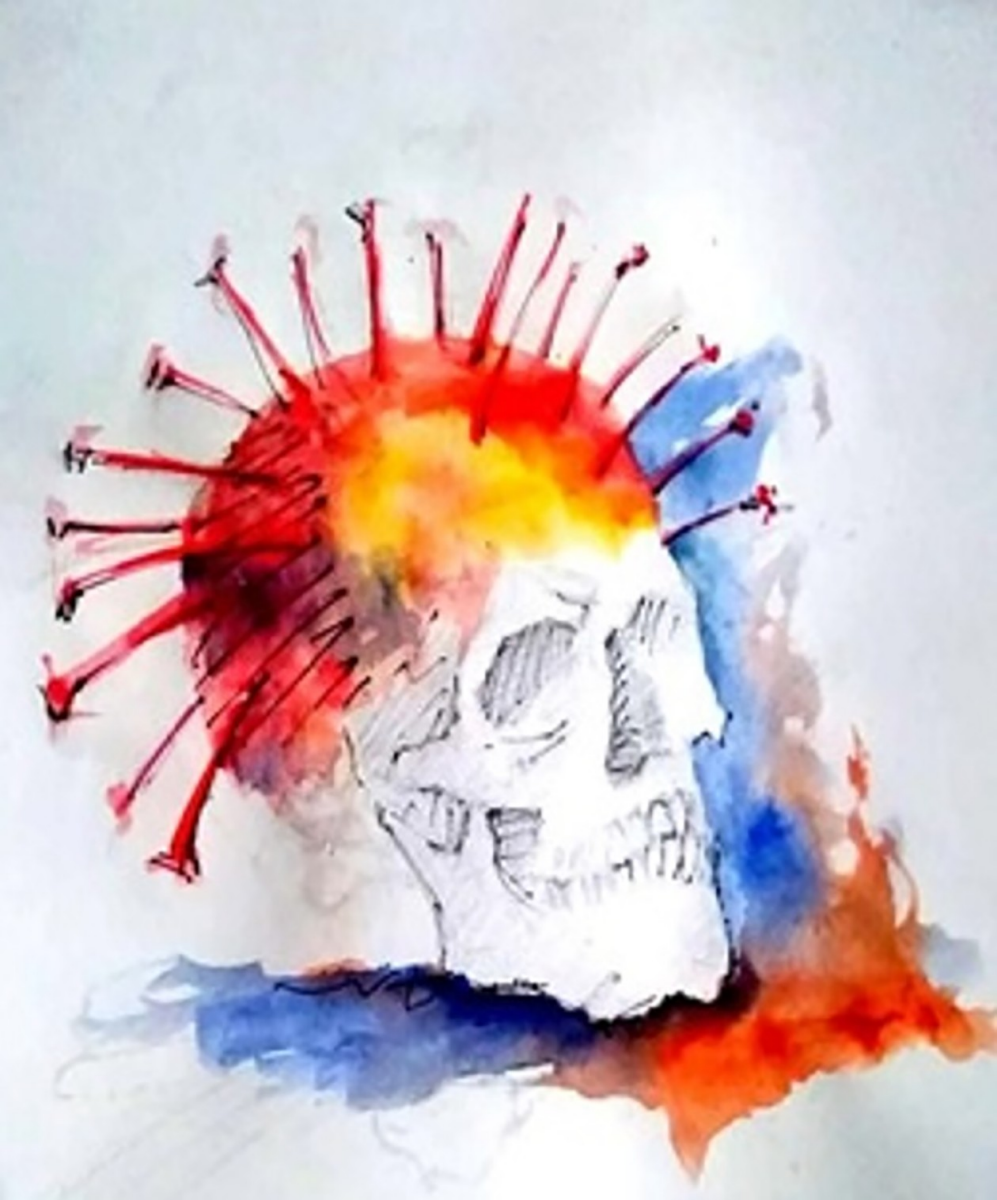
Illustration “fear of being a victim of COVID-19” (2).

The constant fear of acquiring the disease produces an innate need to be relentlessly alert and defensive. This disease is affecting every single person physically, mentally, and emotionally. In Fig 12, one participant illustrated COVID-19 as death waiting beside him with a stopwatch just waiting for any mistake that might make him a victim and engulf him, while the medical personnel are trying to save lives and bring hope to the world. Another participant (Fig 13) labeled coronavirus as the face of death, which could be anywhere, ready and waiting to take you. According to him, we cannot defeat it, but we need to save ourselves by taking as many precautions as we can.

**Fig 14:**
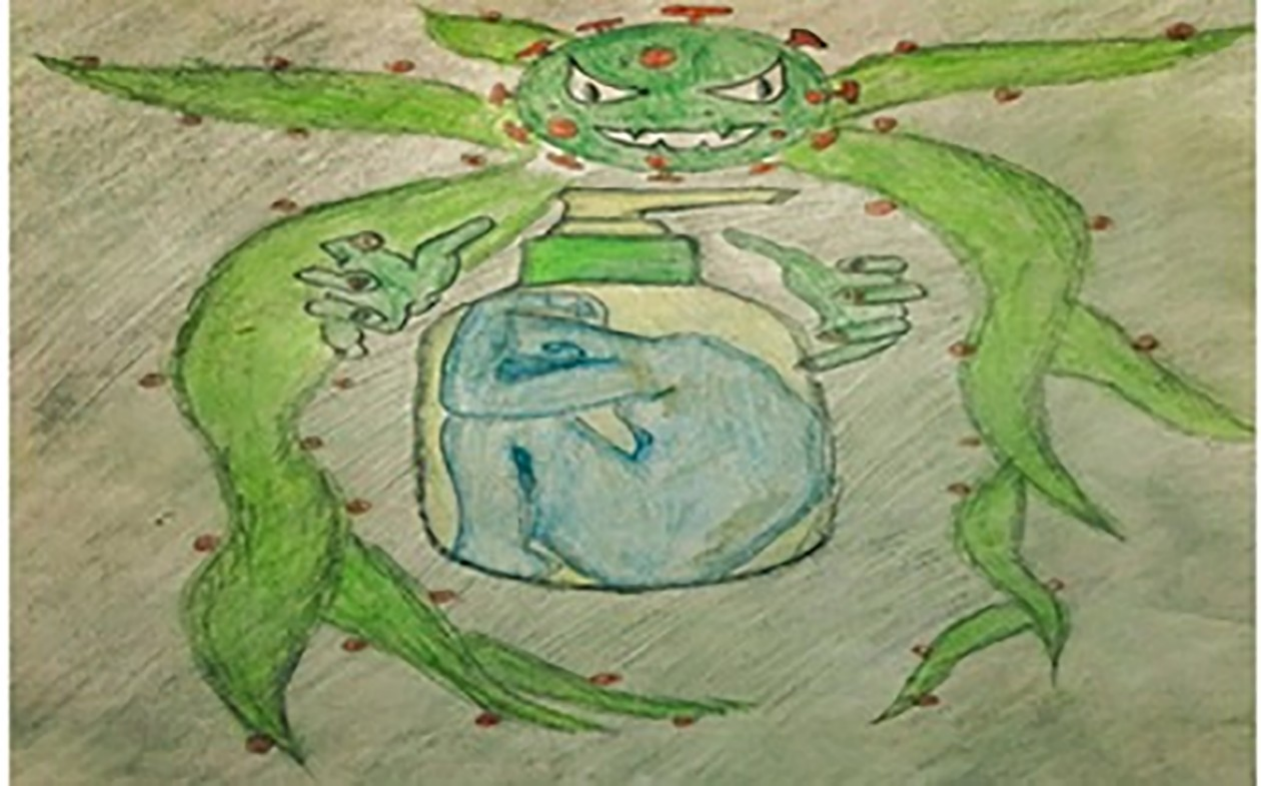
Illustration “fear of being a victim of COVID-19” (3).

**Fig 15:**
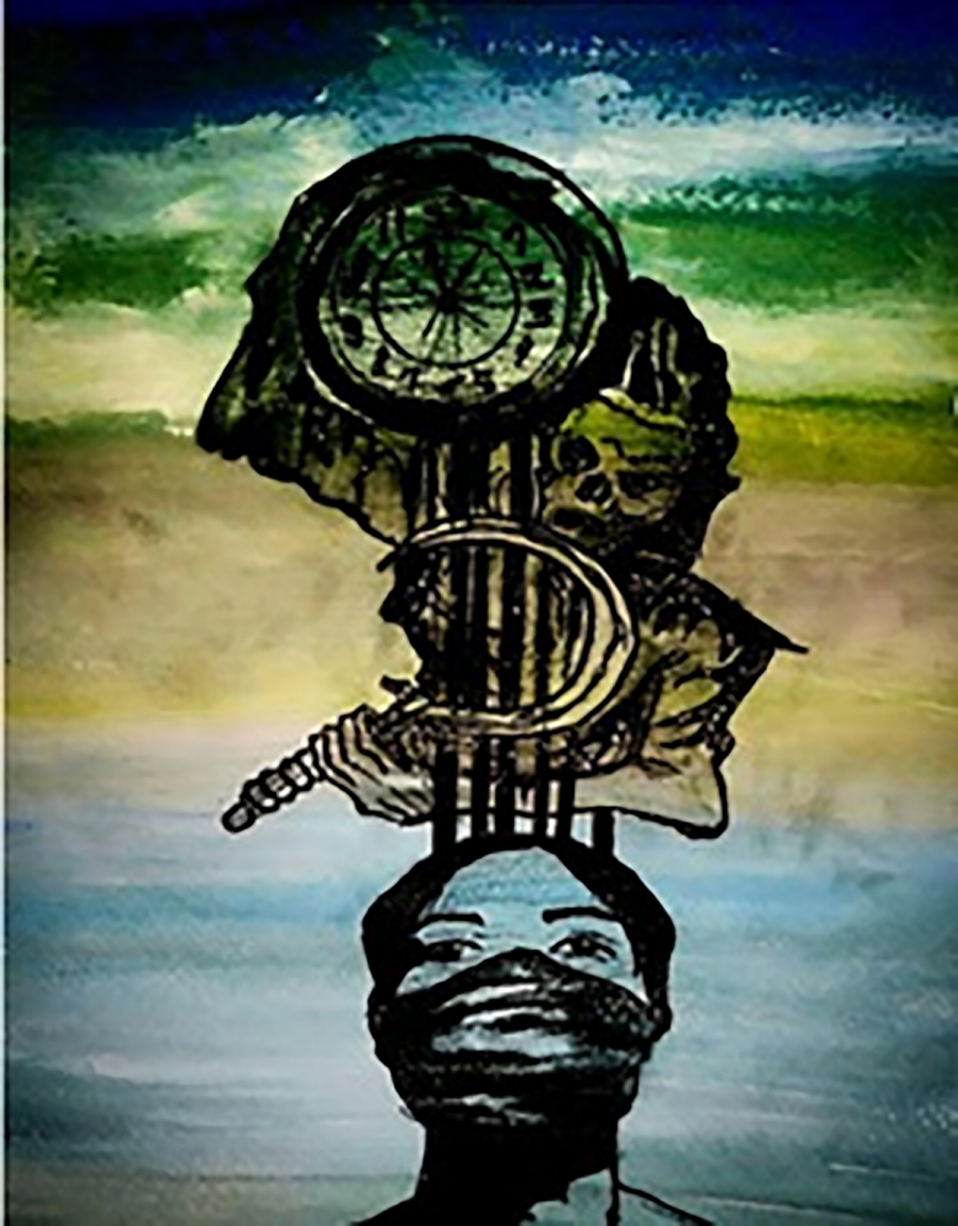
Illustration “fear of being a victim of COVID-19” (4).

**Fig 16:**
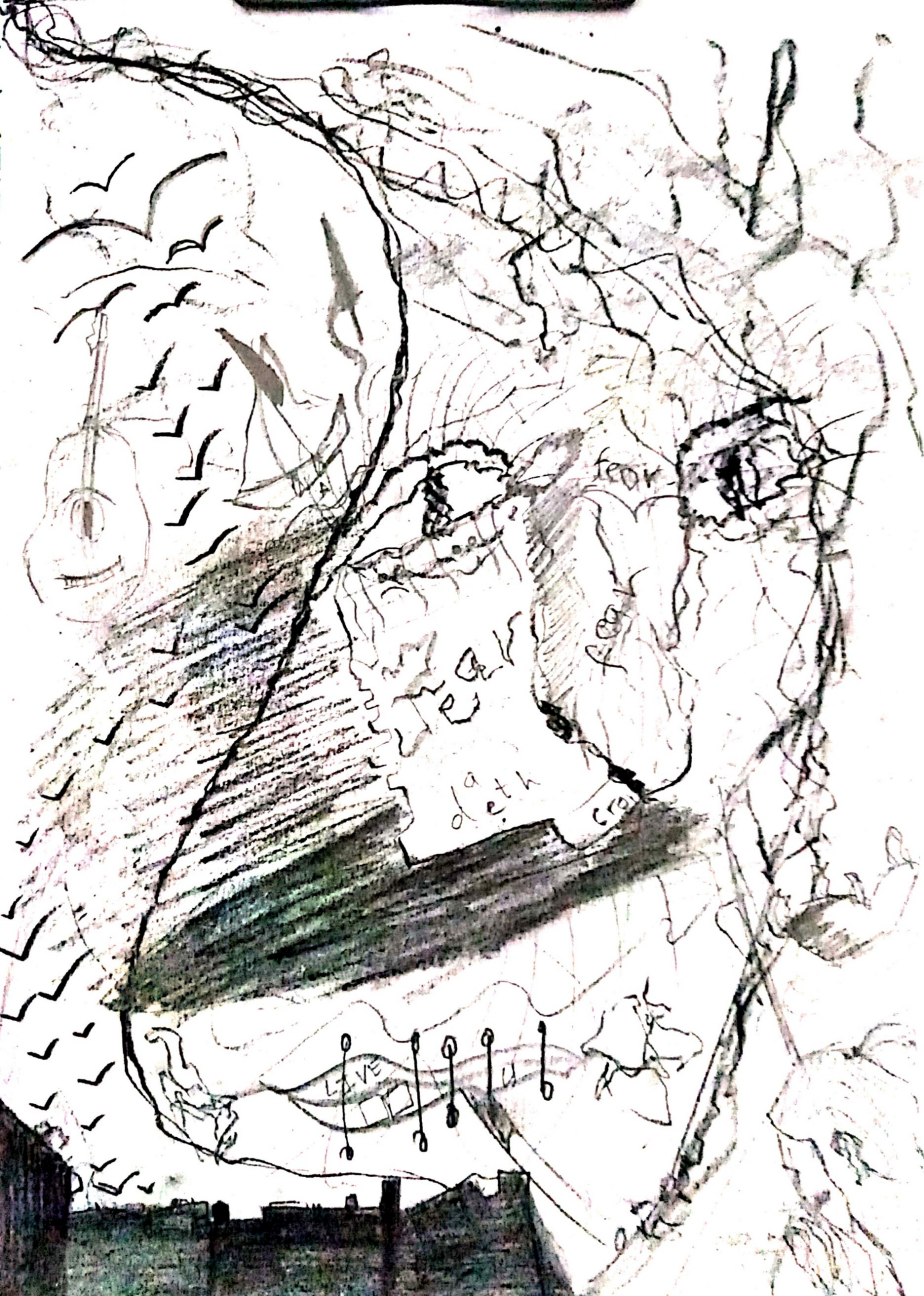
Illustration “fear of being a victim of COVID-19” (5).

Fig 14 depicts the emotions of a participant who feels as though he is enclosed in a chamber (quarantined), illustrated in form of a soap dispenser as a symbol of hygiene, one of our only protections. Outside this chamber, coronavirus has wrapped up everything, which has made life inside more depressing. In Fig 15, the participant shows that clocks are attached to our brains; death is perching on our shoulder all the time. It just seems to be a matter of time before the clock will strike the hour of our demise. Surviving a deadly pandemic reminds us of how brittle life is in this damaged world. Another study participant claims through Fig 16 that it is not the virus itself, but the fear of death that is creating chaos in our subconscious minds. We are not victims of COVID-19; rather, we are victims of the fear that controls all our actions and desires. Therefore, it is time to acknowledge the fear and learn to make our own decisions about survival.

Most of the participants under this theme took a passive coping approach due to the negativity spread through social media. The main cause of depression and poor psychological adjustment was not the pandemic itself, but the fear of death related to COVID-19 that was portrayed on social media.

### Concerns regarding education, future career, and opportunities

**Fig 17:**
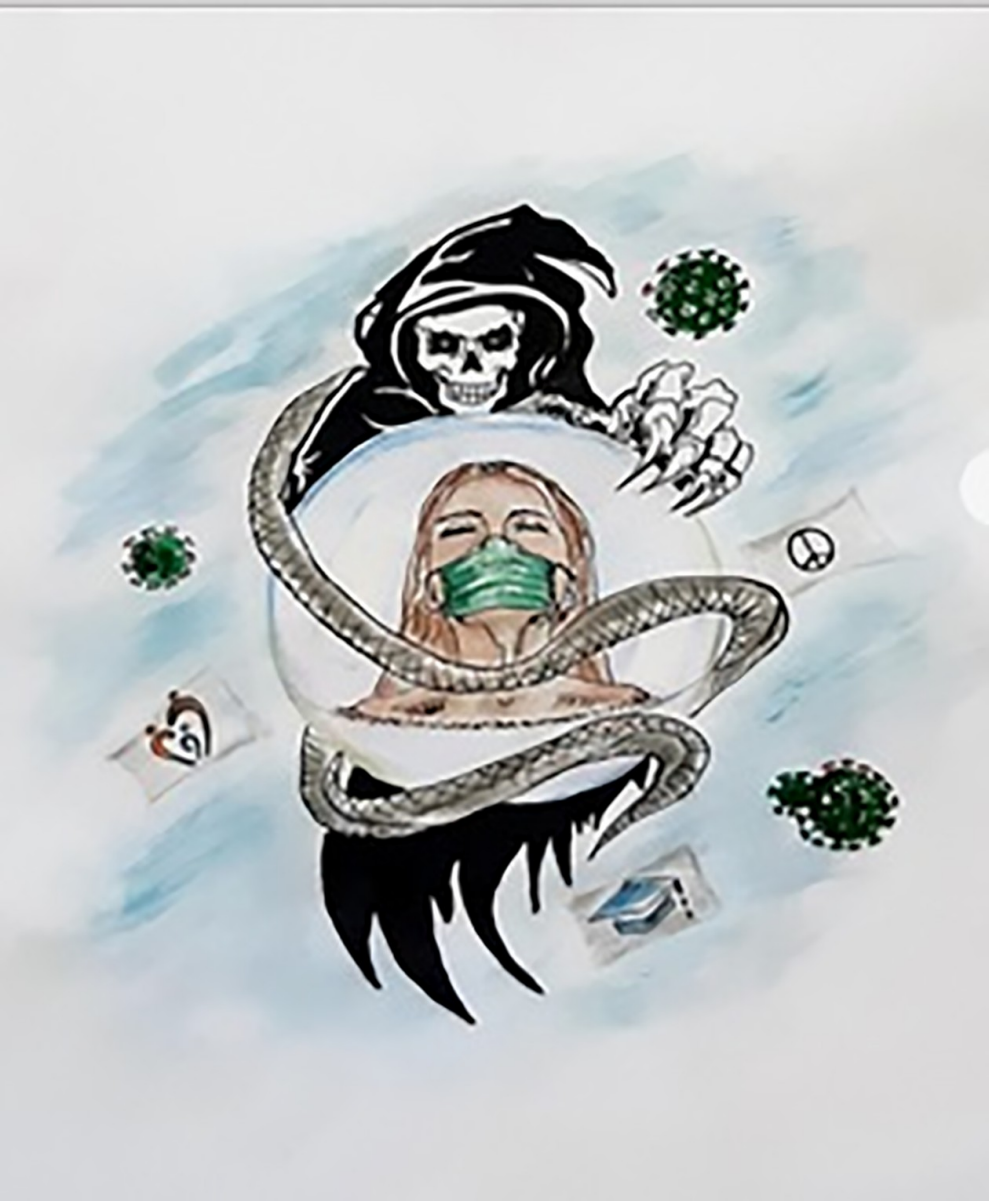
Illustration “concerns regarding education, future career, and opportunities” (1).

Fear of an uncertain future was the major concern for most of the students. This pandemic has brought a halt not only to education, but also to practical life. Fig 17 shows a girl enclosed in a bubble of isolation, the only thing that can keep her safe. If she tries to escape to fulfil her dreams, death will be there waiting for her. Her wishes and desires are lost in fear, which puts education, peace, relationships, and career all in jeopardy.

**Fig 18:**
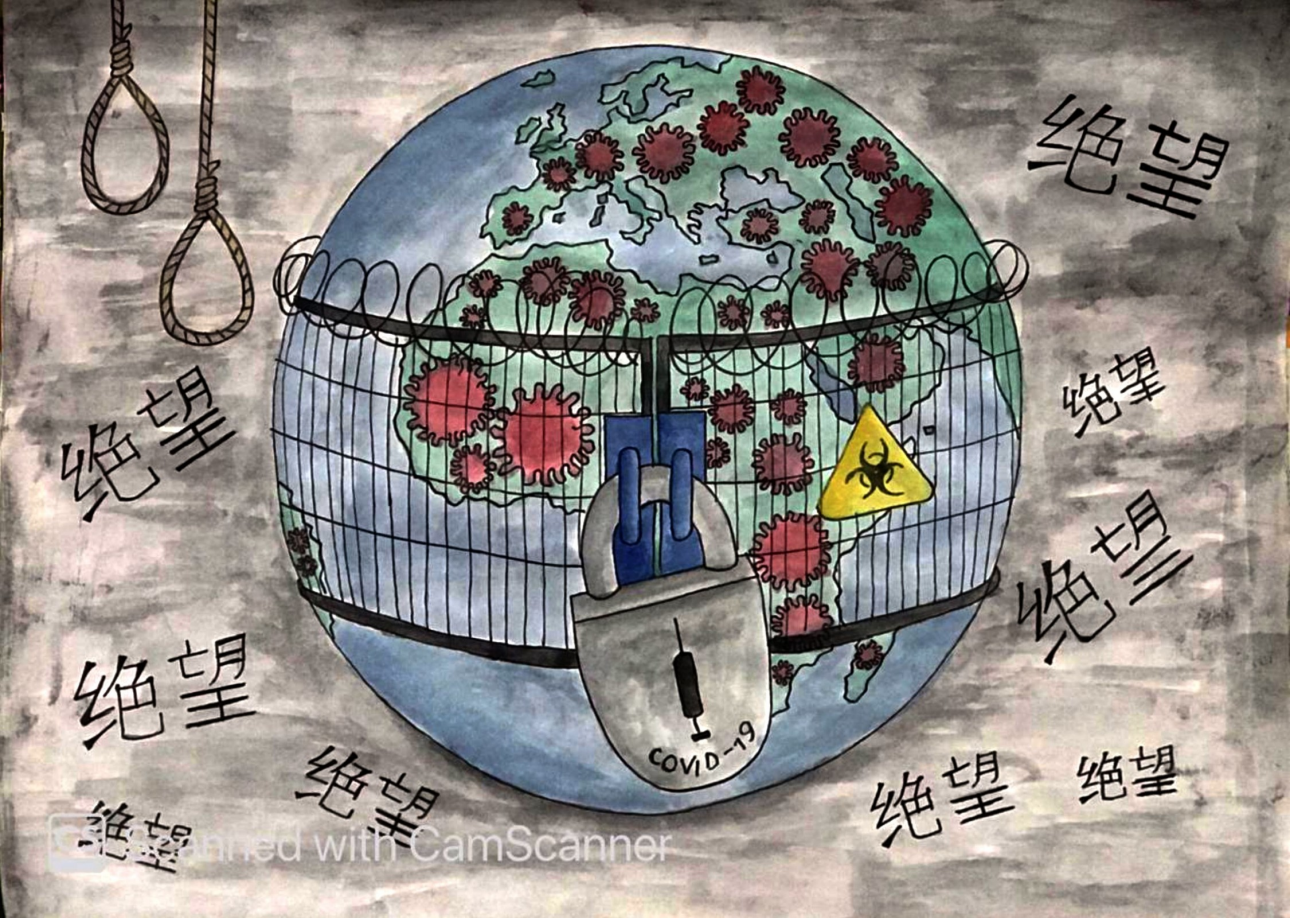
Illustration “concerns regarding education, future career, and opportunities” (2).

Another illustration (Fig 18) shows that the world is locked down due to this deadly virus. Death is hanging everywhere, not only due to coronavirus but also fear of death itself (thanatophobia) is causing people to die. Not only people, but also their dreams, jobs, careers, and the economy are locked down, and all we can see is darkness everywhere.

**Fig 19:**
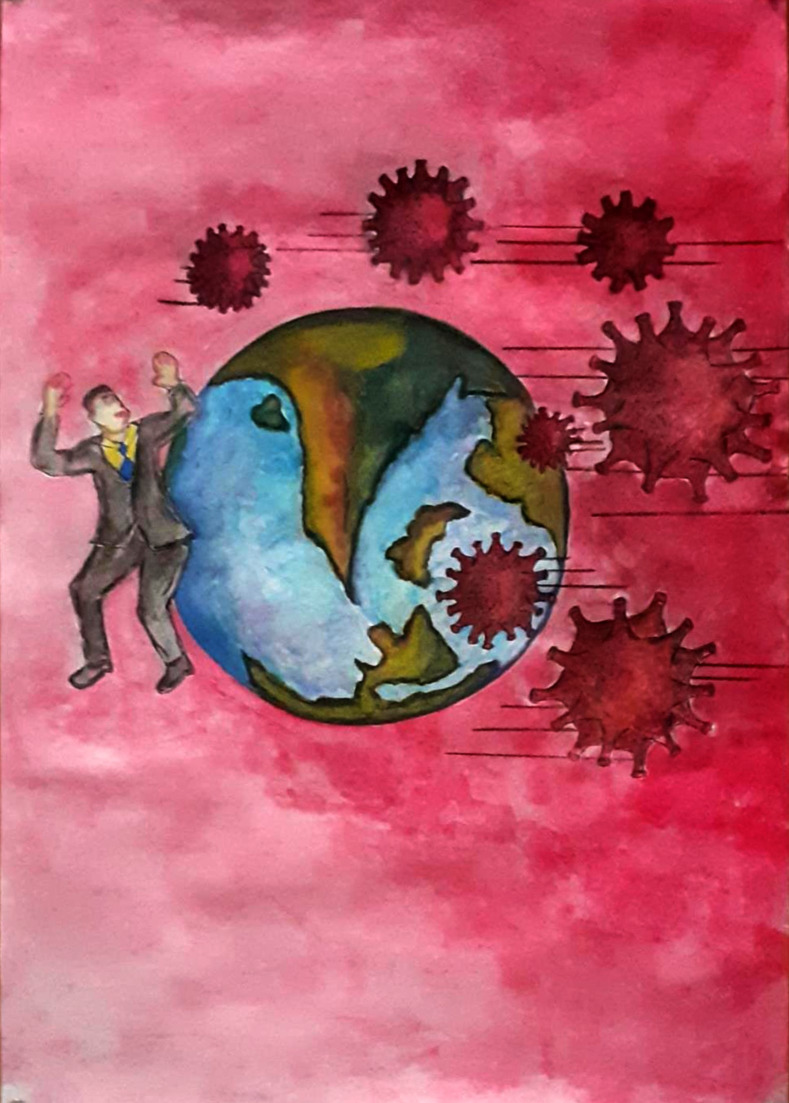
Illustration “concerns regarding education, future career, and opportunities” (3).

**Fig 20:**
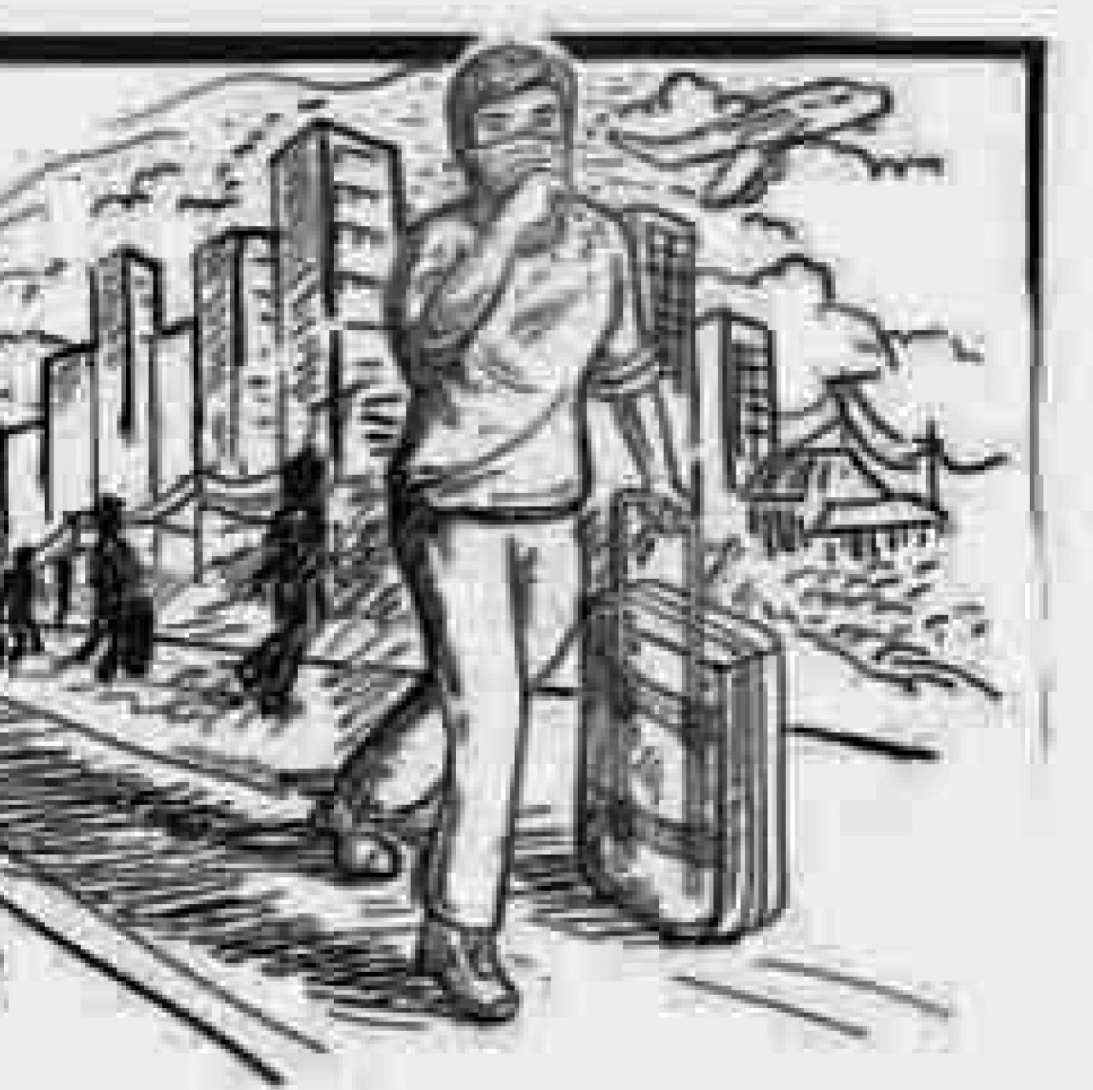
Illustration “concerns regarding education, future career, and opportunities” (4).

In Fig 19, the participant is trying to show that coronavirus has ruined everyone’s career, education, and future. The person shown in the figure represents a well-educated and career-oriented person whose life has been turned upside down. He is trying to save himself, but soon he will be crushed by the cursed world. Another picture (Fig 20), illustrates students and workers from abroad being forced to leave their institutions and jobs due to this pandemic. They have no idea when they will be able to return to their jobs and institutions, or even if they will ever be able to go back due to the economic pressures that coronavirus has generated. This depicts an uncertain future.

**Fig 21:**
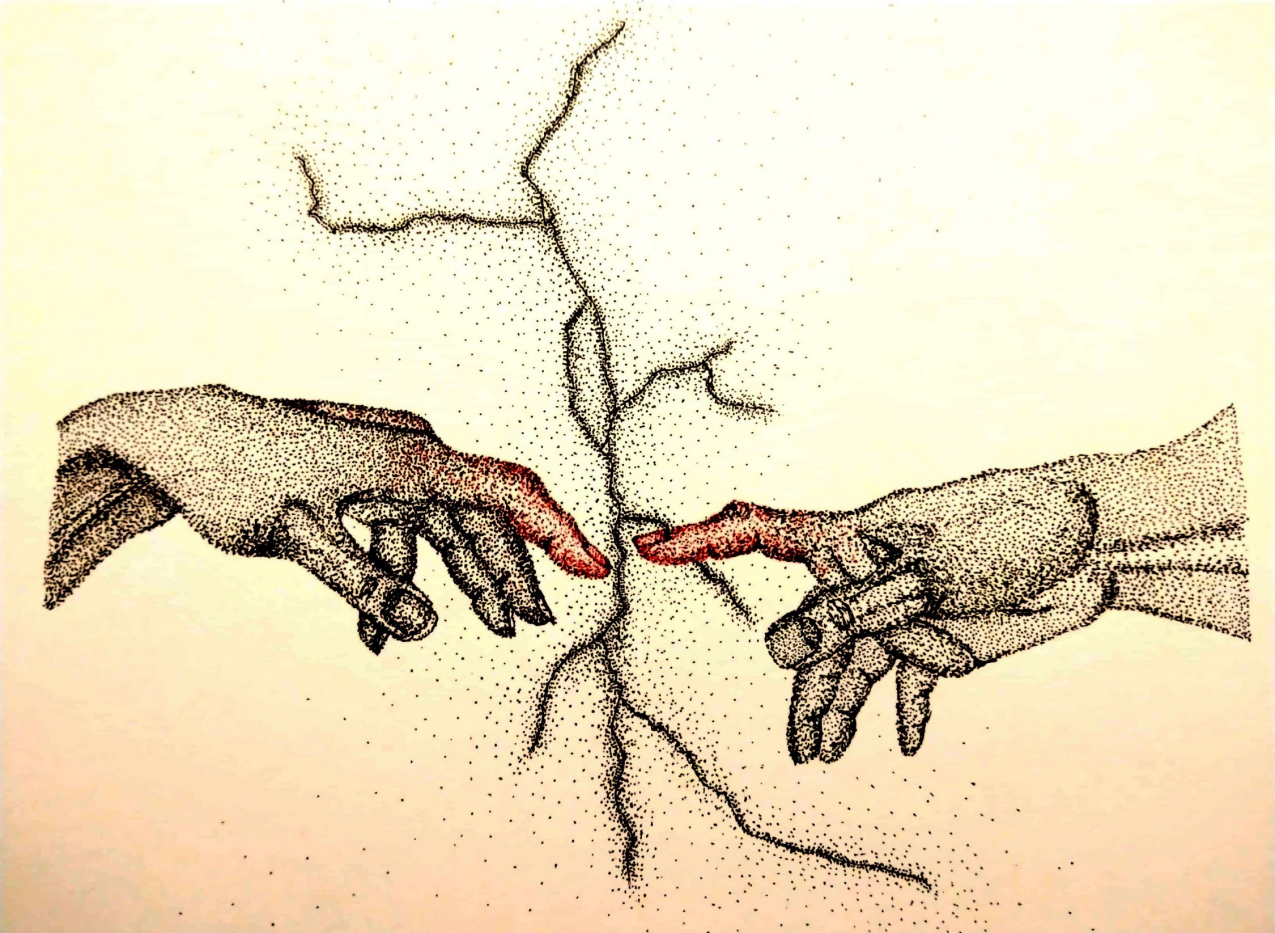
Illustration “concerns regarding education, future career, and opportunities” (5).

Another participant (Fig 21) stated:

“As this pandemic has kept us away from our goals, my illustration depicts invisible walls between a person and his goals. The more we try to move towards our set goals, the more we are exposed to danger. The red color represents danger, which is getting stronger as we try to break down the physical distance between one another.“

**Fig 22:**
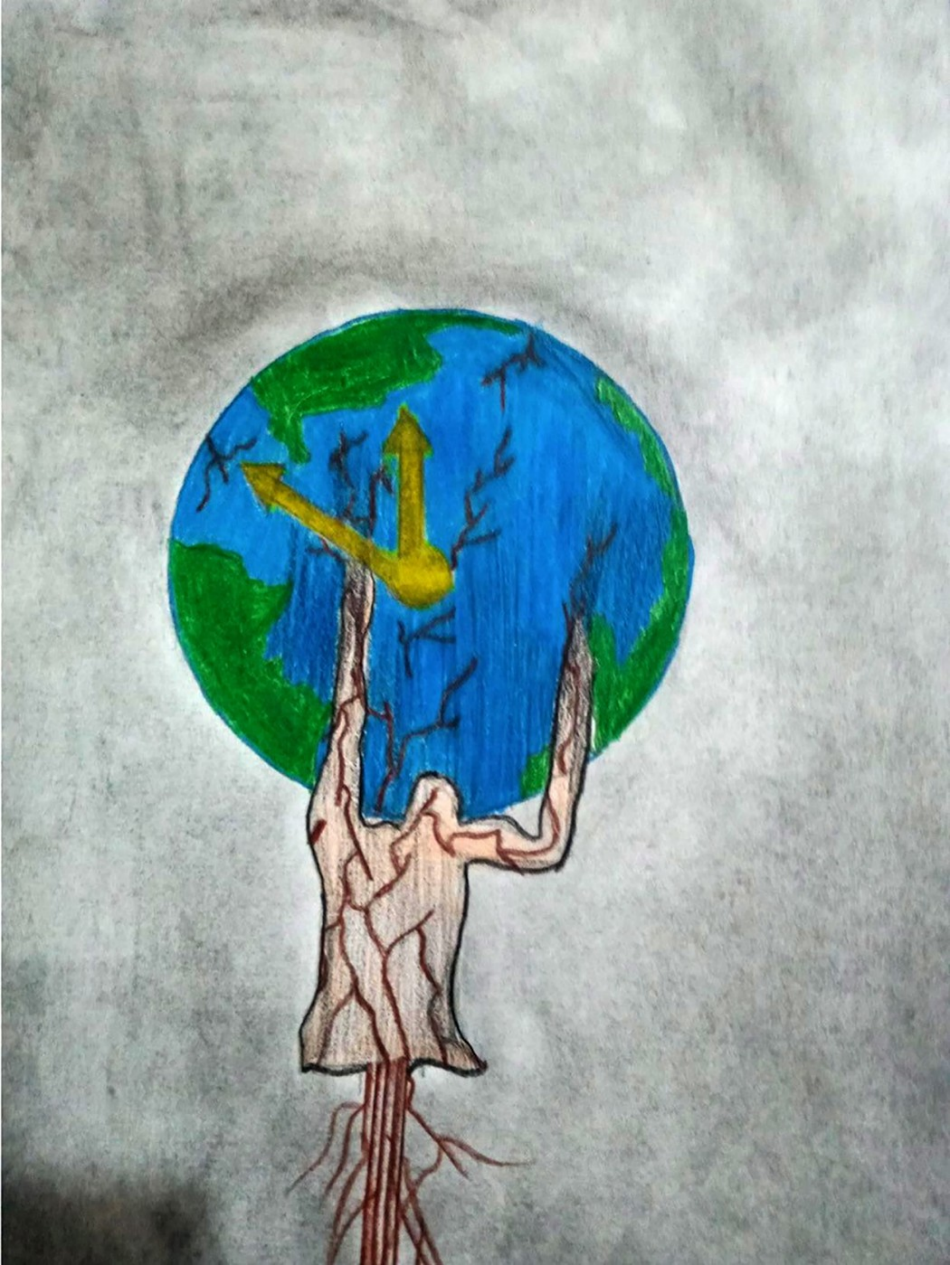
Illustration “concerns regarding education, future career, and opportunities” (6).

Likewise, Fig 22 shows that the world has thrown away people and their dreams. With each passing minute, people are moving further away from their goals. The background represents the anxiety and depression that human beings are experiencing.

**Fig 23:**
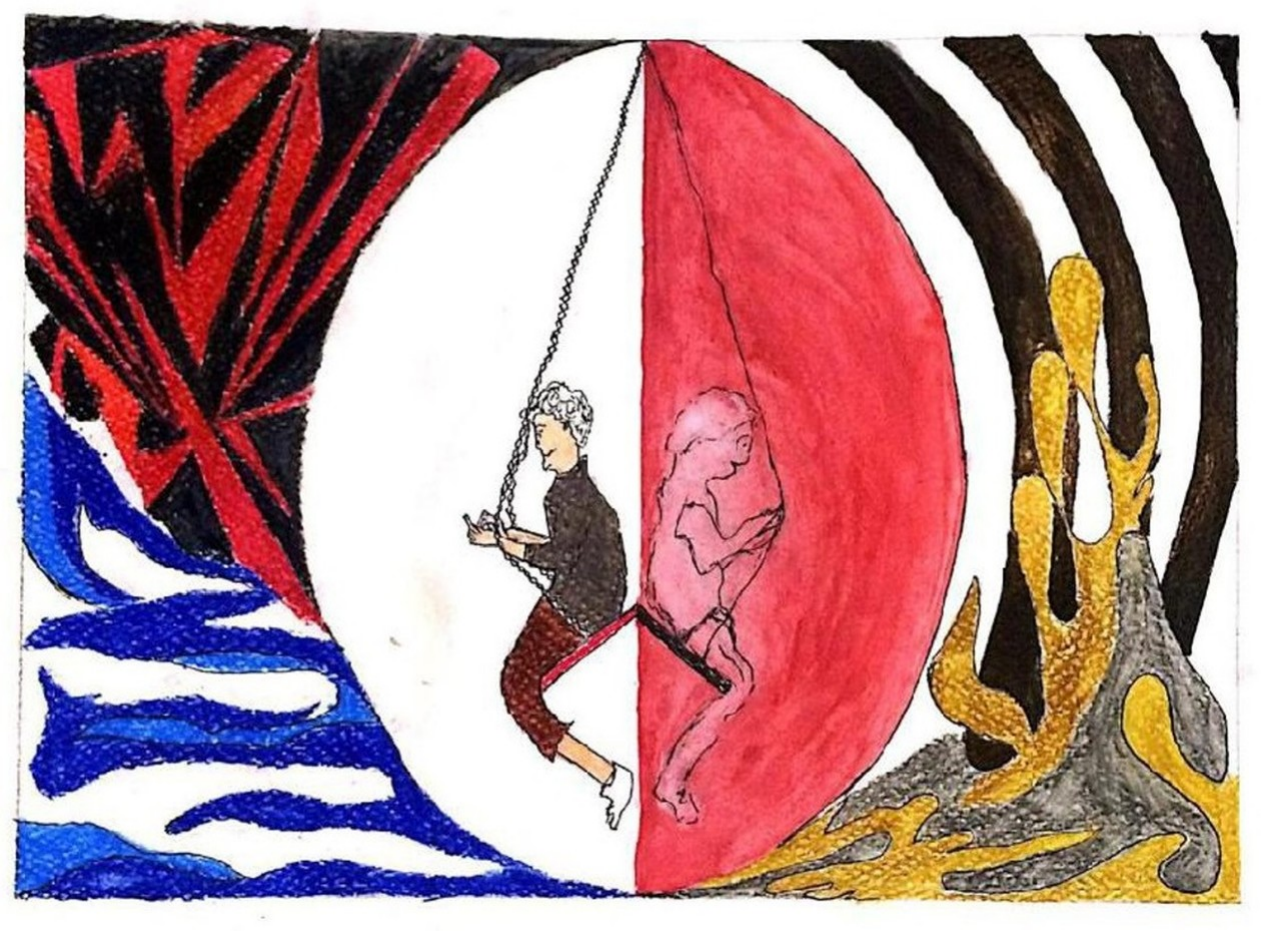
Illustration “concerns regarding education, future career, and opportunities” (7).

The next participant (Fig 23) tried to portray how humans have been living luxurious lives. We had assumed that nowadays mankind could achieve anything, but COVID-19 has proved us wrong. The blue waves in the illustration show the previous success and peace, which are then disturbed by the black and red lines of death and pandemic. The doodle underneath represents the uncertainty about how life will be after this pandemic, while in the center the eclipse and swing show the ups and downs of mankind, starting from the right-hand one, successful and unbeatable, swinging to the left-hand one, which is a fragile, fearful, and unanticipated future.

**Fig 24:**
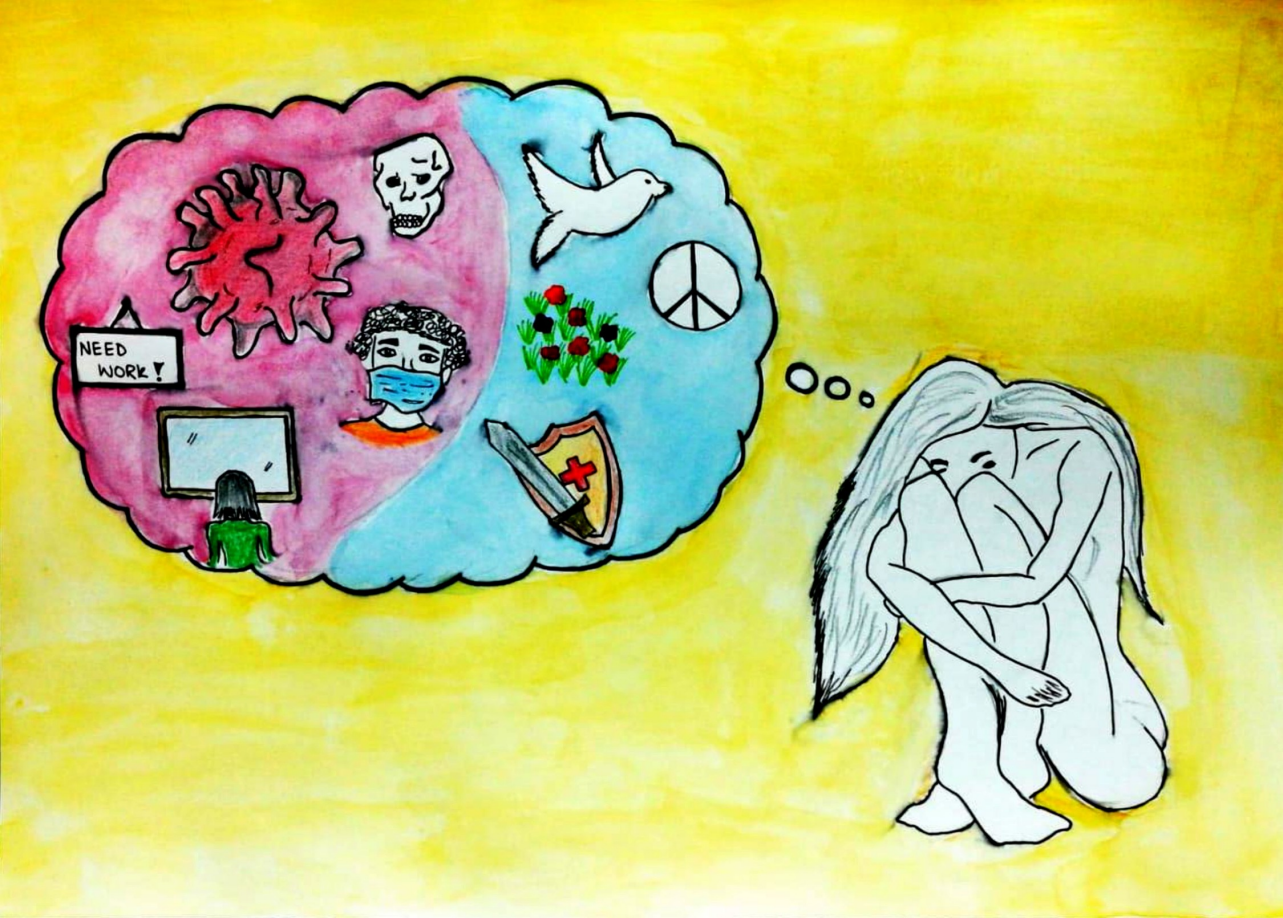
Illustration “concerns regarding education, future career, and opportunities” (8).

**Fig 25:**
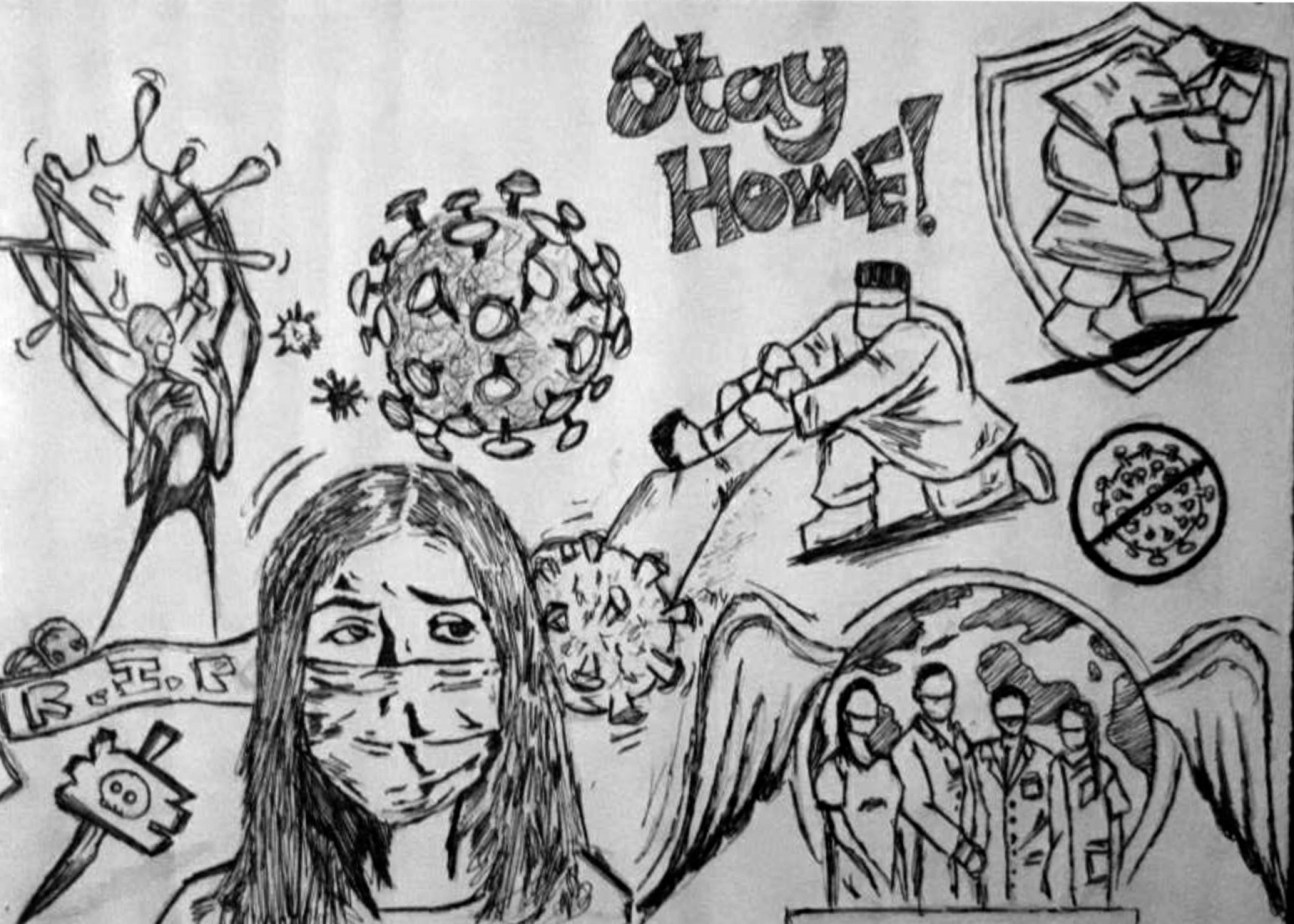
Illustration “concerns regarding education, future career, and opportunities” (9).

Similarly, in the next illustration (Fig 24) the participant portrays a fearful girl thinking about all the worst things that could happen, such as losing her job or her life. Fear of becoming a victim of coronavirus, in terms of health or economics, is ruining her peace of mind. The final illustration (Fig 25) represents the havoc this world is facing. This is not only in terms of excessive deaths, but also compromised education, jobs, business and life goals, and mental and physical health as well as peace. While health-care staff are considered the only frontline warriors, in reality everyone who is suffering from lack of determination, a confusing future, a drowning business or career is fighting this pandemic at the front line.

A majority of participants had concerns regarding their education and future career due to increasing depression, loss of social contacts, and economic crises at home. According to them, these issues were creating a pessimistic approach towards the pandemic and lockdowns.

### Outcome space

**Fig 26:**
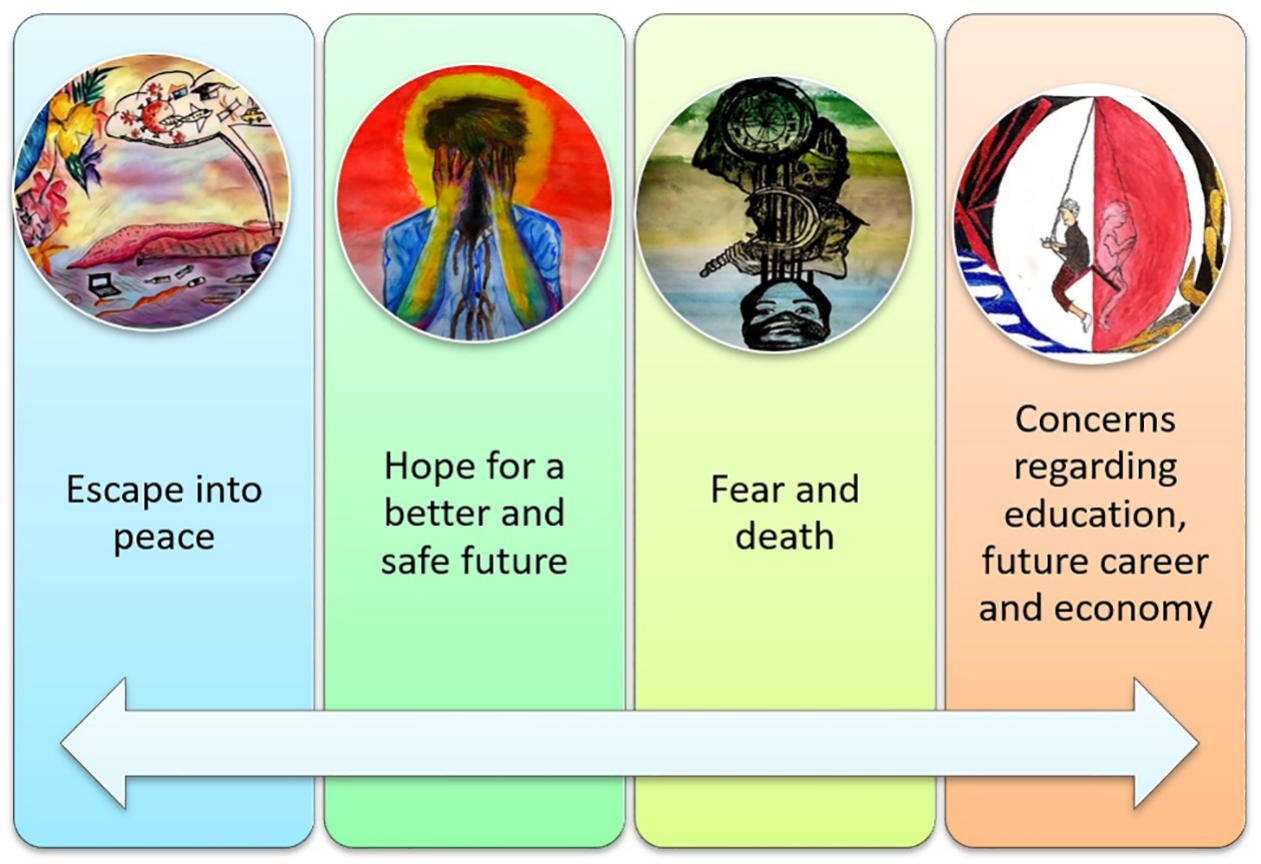
Outcome space.

An outcome space is a graphic representation of the coherent relations among different ideas or notions defined by a study [20]. The outcome space of this research consists of the creative expressions produced by the participants contained within circles focusing on the linkage and dissociation among different meanings of experience [20, 21]. The variation among understandings reveals different ways of thinking and absorbing life experiences among university students. The different themes described portray the unanimity and variances among understandings. They are all experienced at once, instead of in a hierarchical process, which comprises of one classification leading to a new one. The understanding of the world could be defined as existing in an absurdity of “hope or escape to a normal life”, where “forestalling death” or a “compromised future” occur simultaneously. The graph highlights the associations among the classifications/themes, apprehended by the collected data which depicts hope about regaining the pre-pandemic life, fear of becoming a victim of the coronavirus, of losing loved ones, being quarantined away from the people you care about, and being helpless. The arrow is intermixing and moving between the themes as the enormity of life and hope is interlinked with the expectation of death, which is always present and constantly nearby. The fear of death is enormously increased due to the unstable situation, growing depression, enhanced social media discussions, and lack of physical interaction due to the lockdowns. For that reason, the outcome space presented in Fig 26 simultaneously represents active and passive ways of coping with the present situation among university students in Pakistan. It starts by nullifying and escaping from the situation while hoping and praying to get back to normal (pre-pandemic), along with the fear of losing loved ones and future dreams. The first block is the escape from present circumstances, starting by considering the situation to be unreal and shifting towards seeing it as nature’s way of rejuvenating and replenishing itself after the destruction humans have wreaked over the years. A few participants also consider this situation to be the Almighty’s way of showing humankind their reality, because all they want is peace for themselves and their loved ones. The second theme is hope, and this depicts scenes of a victim of the virus or lockdown hoping that things will get better. It creates an optimistic approach by trying to value what we had in the past, which we never really valued at the time. This could be personal freedom, healthy times with family or friends, good health, and a safe environment. This theme gives us time to reconsider our priorities, real happiness, and the meaning of life. The third theme is the most depressive of all, and is about the haunting of mental health. It is about fear which makes individuals more prone to psychological impairments. Fear of losing your loved ones, infecting them, or not being able to help them has increased the adverse effects of the pandemic many times over. Social media interactions during lockdown, media reports, and the increasing death toll have led to fear. The next theme is crucial among all the participants, because it revolves around the future, with or without the pandemic. The educational and economic losses will have long-term effects on participants’ lives. The uncertainty of life has made career opportunities scarce, which has made survival difficult for many individuals. However, hope is omnipresent. If we want to comprehend the study under one theme, then that overall theme would be: “Hope for life while paradoxically living with fear”, which links all the themes described above in a meaningful sense.

## Discussion

COVID-19 is having distinct effects on the lives of students, both physically and psychologically, all over the world. It has not only jeopardized their physical, social, and mental health, but also their education, future plans, and job opportunities. Furthermore, it creates insecurity about the health and lives of their loved ones. Several studies have investigated psychological effects such as anxiety, depression, and stress among university students related to the COVID-19 outbreak and lockdown [23–25]. But all of these studies were quantitative in nature, thus they could not portray actual insights into the students’ concerns and feelings as this study has done. A study conducted in Spain demonstrated a moderate to severe adverse impact due to this outbreak, psychologically. These effects were particularly strong in students from the fields of arts, humanities, social sciences, and law compared to students from engineering and architecture [26]. In accordance with this study, the art students in our research, who are strongly affected by the pandemic and lockdown, illustrated their deep-rooted emotional concerns, social seclusion, and remorse over not having cherished their personal freedom, health, and happiness in past.

In the absence of a vaccine, lockdown was necessary in order to control the unknown and extremely infectious COVID-19 pandemic [7]. This intervention, however, has forced healthy people to suffer the adverse effects of lockdown as well [27]. Although education and business have continued online to some extent, this is nowhere near the level of actual interactive education or trade. This leads to multiple psychological impacts of lockdown. These adverse effects are more pronounced among students in developing countries [28] due to scarce socioeconomic resources.

The analysis of the illustrations created for this study revealed four different but interconnected approaches to managing the emotional turmoil of lockdowns related to COVID-19. As in other studies, a few of the participants had hope and faith that was stronger than average, while others were more pessimistic about the ongoing situation. Overall, the findings were psychologically more or less similar. They included fearfulness, isolation, apprehension, insomnia, boredom, resentment, annoyance, ambiguity, and stigmatization. Experiencing the lockdown was not stress-free for these university students. They were combating social distancing as well as the turbulent economic and health circumstances their families were facing. Similar to other studies, quarantined participants had doubts about the actual situation due to the hype created within the media and were hesitant about accepting this lockdown as the most effective technique for combatting the pandemic [6].

This study has given us an opportunity to understand the requirements, hopes, and thoughts of young people who are facing these drastic infection-control strategies. Several illustrations undoubtedly depict an appreciation of the worth of living a free and peaceful life, which we took for granted before. Furthermore, the unpredictability of COVID-19 and its enduring effects elevate feelings of improbability and uncontrollability in relation to the future. This augments the sense of agony and helplessness which is evident in the illustrations [4]. COVID-19 has made us realize with trepidation that we could die from a dreadful disease which is indifferent to all of our values, dreams, and pride [29]. The results of this study could help in gaining insight into the psychological issues that university students are experiencing during lockdown. This could support the devising of effective and comprehensive strategies related to education, emotional counselling, and stress management for improving the mental health of students. Providing adequate information, including the motives for introducing lockdowns and their usefulness for the general public, could reduce the adverse psychological impact of such lockdowns.

### Limitations

Our results need to be interpreted with caution due to some limitations. First of all, the sample limits the diversity of understandings that the human mind could relate to a specific situation. Nevertheless, the sample is quite large for such a qualitative study. There have been no further inclusion or exclusion criteria, despite the fact that they were students of arts and design. There was no need to exclude students who seemed to be depressed, although we did not assess their current state of mental health in a standardized way. Including depressed students may have influenced the results, because persons with depressed mood articulate their emotions and thoughts differently in illustrations. The topic of anxiety and depression was frequently addressed in the illustrations. The interpretation would have been different in a sample of students with depression. The choice of only arts students at one university limits their areas of concern. It needs to be kept in mind that students from other disciplines could have different understandings. The diversity related to age groups and professions could not be delineated.

## Conclusions

This study highlights the psychological impact of the COVID-19 pandemic on students. The qualitative design allows insights into the psychological effects experienced by students during lockdown. The themes, related to fears (such as becoming a victim of COVID-19), concerns (e.g., in terms of education and future career opportunities), as well as the hope for personal freedom and an escape into peace, emphasize the need for supportive programs for relieving stress. Students need to be better prepared to face and overcome the long-lasting gaps this pandemic has created in terms of economic, educational, and career opportunities.
